# Uncommon C18 Conjugated Dienes Define the Sex Pheromone System of *Thelosia camina* (Lepidoptera: Apatelodidae), a Pest of Yerba Mate

**DOI:** 10.1021/acs.jafc.5c14499

**Published:** 2026-01-04

**Authors:** Diogo M. Vidal, Emir B. Saad, Miryan D. A. Coracini, Rafael C. G. Pereira, Marcílio J. Thomazini, Carme Quero, Maria P. Bosch, Ángel Guerrero, Paulo H. G. Zarbin

**Affiliations:** † 425909 Department of Chemistry, Universidade Federal de Minas Gerais, Av. Antônio Carlos, 6627, Belo Horizonte-MG 31.270-901, Brazil; ‡ Department of Chemistry, 28122 Universidade Federal do Paraná, Av. Cel. Francisco H. dos Santos, 100, Curitiba-PR 81.531-990, Brazil; § Biological Sciences and Health Center, UNIOESTE, Rua Universitária, 1619, Cascavel-PR 85.819-110, Brazil; ∥ Embrapa Florestas, BR-476, km17, s/n, Colombo-PR 83.411-000 Brazil; ⊥ Department of Biological Chemistry, 203230 Institute of Advanced Chemistry of Catalunya-CSIC, Jordi Girona,18-26, Barcelona, 08034, Spain

**Keywords:** conjugated dienes, electroantennographic detection, yerba mate pest management, lepidopteran chemical communication, sex pheromones

## Abstract

The yerba mate tree *Ilex paraguariensis* is widely consumed in South America, and the limited recommendation for pesticide use highlights the need for alternative pest-management strategies. *Thelosia camina* is an important defoliator whose damage severely reduces yields. This study investigated the sex-pheromone system of *T. camina* as a basis for pheromone-mediated control. Female pheromone glands were extracted and analyzed by gas chromatography-electroantennographic detection, revealing nine antennally active compounds. Structural elucidation identified three major components (13*Z*,15*Z*)-octadeca-13,15-dienal (**4**), (13*Z*,15*Z*)-octadeca-13,15-dien-1-ol (**5**) and (13*Z*,15*Z*)-octadeca-13,15-dien-1-yl acetate (**9**). Remaining components were stereoisomers and related monoenes consistent with the same double-bond system. All isomers were synthesized, field bioassays showed that both the ternary blend (**4**,**5** and **9**) and the acetate (**9**) alone attracted males at levels significantly higher than controls. These results confirm conjugated C18 dienes as key pheromone components of *T. camina* and provide a foundation for pheromone-based, sustainable management in yerba mate cultivation.

## Graphical abstract

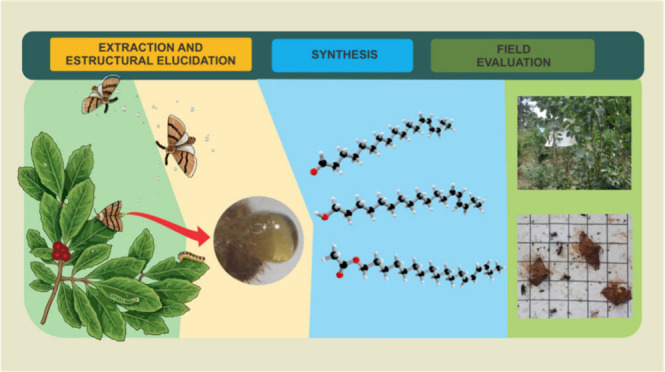

## Introduction

Insect pests remain a major challenge for crop production worldwide, with their impact becoming increasingly critical over the past century.^1^ In Brazil, one of the world’s leading agricultural producers, average annual yield losses are estimated at approximately 7.7%, despite the adoption of a variety of pest management strategies.^2^ Within this context, yerba mate (*Ilex paraguariensis* A. St.-Hil) represents a crop of significant socioeconomic relevance in southern Brazil and neighboring countries, such as Argentina, Paraguay, and Uruguay, where it is the raw material for *chimarrão* and other popular caffeinated beverages.^3^ Production is concentrated in the state of Paraná, placing Brazil as the leading global producer. Cultivation in the region predominantly occurs in family based agroforestry systems, acknowledged by the FAO as a Globally Important Agricultural Heritage System (GIAHS) due to their cultural, social, and environmental significance.^4^

One of the main constraints on yerba mate production is *Thelosia camina* (Schaus, 1896) (*T. camina*; Lepidoptera: Apatelodidae), a moth whose larvae feed voraciously on the leaves, leading to severe defoliation that reduces yields and weakens the trees.^5^ Under high infestation levels, entire branches or plants may be defoliated, directly affecting both the quantity and quality of harvestable leaves.^6,7^ Beyond these agronomic effects, infestations can cause long-term physiological stress, reducing photosynthetic capacity, vigor, and plant longevity. Although direct financial losses attributable solely to *T. camina* have not yet been quantified, severe outbreaks of yerba mate pests have been associated with significant yield reductions and increased production costs.^8^ For comparison, biological control initiatives targeting other major pests of yerba mate, such as *Hedypathes betulinus*, have been estimated to prevent productivity losses of up to 20%, representing an economic benefit of approximately BRL 4.36 million for the yerba mate production chain.^9^

The synchronization of larval population peaks of *T. camina* with the leaf harvest period, together with the small-scale agroforestry production system, renders conventional chemical control unfeasible, as pesticide use would pose a high risk of contamination in the harvested leaves. In this scenario, semiochemical-based strategies offer a promising and environmentally acceptable alternative. We, therefore, focused on the sexual pheromone of *T. camina*, aiming to elucidate its chemical structure, synthesize candidate compounds, and evaluate their biological activity. Given that our research group has previously identified and synthesized the male-produced aggregation pheromone of the main coleopteran pest of yerba mate, *Hedypathes betulinus* (*H. betulinus*; Coleoptera: Cerambycidae),^10–13^ and studied the response of yerba-mate plants toward *H. betulinus* and *T. camina* herbivory,^14^ the present identification of the sex pheromone of *T. camina* represents an important complementary advance. Together, these findings pave the way for the development of an integrated, pheromone-based management strategy targeting the two principal insect pests of yerba mate, thereby promoting environmentally sustainable and residue-free control approaches for this culturally and economically important crop.

## Methods and Materials

### Insects

Insects in the pupal stage were collected at Embrapa Florestas experimental fields, Colombo, Paraná, Brazil (25.32°S, 49.15°W). They were collected from the soil beneath *Ilex paraguariensis* (*I. paraguariensis*) trees that presented catterpillars infestation simptoms on the leaves. At the end of the larval instar they go to the soil at the base of the plant stem to pupate. To collect the pupae, the ground vegetation under selected trees was carefully removed, and the soil was gently excavated to a depth of approximately 10 cm. The excavated soil was then sieved to isolate the pupae. The pupae were kept in the laboratory to get the adults to initiate the laboratory rearing. The rearing was conducted under controlled conditions (24 ± 1.0 °C, with 70 ± 10% relative humidity, and L:D 14:10 photoperiod). After emergence, larvae were fed with fresh yerba mate (*I. paraguariensis*) leaves obtained from trees without pesticide application. The fresh leaves were treated with a 1% sodium hypochlorite solution to prevent microbial contamination. As the larvae developed through instars and increased in size, they were transferred to 1 L plastic containers and later to 10 L plastic boxes (12 × 26 × 36 cm^3^) covered with nylon mesh lids. At the beginning of each scotophase, adults were collected and sexed according to the method described by Thomazzini.^5^ Some adults were individually separated in plastic containers (7 × 5 × 7 cm^3^) covered with nylon mesh, lined with moistened filter paper and fed with a solution of water/honey at 10% until the moment to proceed with the experiments. Some couples were placed together into 1 L plastic boxes covered with nylon mesh, lined with moistened filter paper, and were fed with a solution of water/honey at 10%. The egg masses obtained from the adults were placed in acrylic boxes (6 × 6 x 3 cm^3^) lined with moistened filter paper, to keep the rearing to get enough insects to do the tests.

### Collection of Volatile Compounds

Pheromone glands were dissected from the terminal abdominal segments of 1- to 5-day-old females during peak calling activity (last hour of scotophase to first hour of photophase). Each gland group (5 to 10 glands per group) was placed in a glass vial with 10 μL of distilled hexane per gland and extracted for 20 min. The solvent was then removed via micropipette, transferred to a sealed vial, and stored at −20 °C for subsequent analyses.^15^

### Chemical Analyses

Crude extracts were analyzed by Gas Chromatography coupled with Mass Spectrometry (GC-MS; Shimadzu QP-2010 Plus, EI 70 eV) using a DB-5 capillary column (30 m × 0.25 mm i.d., 0.25 μm film thickness) (Agilent, Santa Clara, CA, USA) with helium as carrier gas (1 mL/min). An aliquot of 1 μL was injected in splitless mode at 250 °C. The oven temperature was held at 50 °C for 3 min, increased at 7 °C/min to 250 °C, and held for 10 min. The transfer line was maintained at 270 °C. Extracts were also analyzed by gas chromatography coupled with Fourier transformed infrared spectroscopy (GC-FT/IR; Shimadzu GC-2010 coupled to a DiscovIR-GC detector; 4000–750 cm^–1^, 4 cm^–1^ resolution) (Spectra Analysis, Marlborough, MA, USA), using the same column, injection mode, and oven program as above. Gas chromatography coupled with electroantennographic detection (GC-EAD) analyses were performed using a Shimadzu GC-2010 coupled to a Syntech electroantennographic detector (Hilversum, Netherlands). The system was equipped with a DB-5 capillary column and operated in splitless mode at 250 °C. The oven was programmed from 100 °C (1 min) to 250 °C at 7 °C/min, with a final hold of 10 min. Electroantennographic responses were recorded using Syntech GC-EAD32 software (v.4.6). To calculate linear retention indices (RI), 1 μL of a stock solution of straight-chain hydrocarbons (C_10_–C_26_) was mixed with 1 μL of the target compound or natural extract and coinjected into the GC. The oven temperature was programmed to start at 50 °C, increase at 3 °C/min to 270 °C, and hold for 10 min. Analyses were carried out on a DB-5 column (30 m × 0.25 mm i.d., 0.25 μm film thickness) as above. Linear retention indexes were calculated following the method of van den Dool and Kratz.^16^ NMR spectra of synthetic compounds were recorded on Bruker ARX-200 and Bruker Avance DRX-400 and DRX-600 instruments using deuterated chloroform as solvent, and tetramethylsilane (TMS) as internal reference.

### Addition of 4-Methyl-1,2,4-triazoline-3,5-dione (MTAD) to Conjugated Dienes

In a 300 μL V-shaped vial, 2 μL of MTAD solution (1% (w/v) in DCM), and 10 μL of the female gland extract were added. After 10 min, the sample was analyzed by GC-MS.^17,18^

### Chemical Synthesis

#### 12-Bromododecan-1-ol (**13**)

1,12-Dodecanediol (**12**) (3.37 g, 16.7 mmol), HBr (2,2 mL, 48%), and toluene (30 mL) were added to a round-bottom flask, equipped with a Dean–Stark, at room temperature. The mixture was refluxed for 6 h, cooled to room temperature and a new portion of HBr (0.5 mL, 48%) was added. The mixture was refluxed for 6 h more and the conversion rate followed by TLC (4:1 hexane/ethyl acetate). After this period, the mixture was diluted in diethyl ether (60 mL), washed with NaOH (1 mol·L^–1^) and brine. The organic layer was dried with anhydrous Na_2_SO_4_, filtered and the solvent evaporated. The crude product was purified by flash chromatography (8:2 hexane/ethyl acetate), affording **13** in 89% yield.^19^ ^1^H NMR (400 MHz, CDCl_3_, ppm): δ 3.62 (t, 2H, *J* = 6.6 Hz), 3.39 (t, 2H, *J* = 6.9 Hz), 1.90 (dq, 2H, *J* = 15.5 Hz, *J* = 7.8 Hz), 1.61 (dq, *J* = 15.1 Hz, *J* = 7.6, 2H), 1.48–1.35 (m, 2H), 1.35–1.23 (m, 14H). ^13^C NMR (100 MHz, CDCl_3_, ppm): δ 63.1, 34.0, 32.8, 32.8, 29.5, 29.5, 29.5, 29.4, 29.4, 28.7, 28.1, 25.7. MS (EI, 70 eV; *m*/*z* (%)): 41 (60), 42 (14), 43 (33), 54 (10), 55 (100), 56 (23), 57 (26), 67 (23), 68 (31), 69 (84), 70 (18), 81 (17), 82 (28), 83 (66), 95 (10), 97 (59), 111 (14), 137 (10), 148 (20), 150 (19), 162 (10), 164 (10), 218 (2), 220 (2).

#### 2-(12-Bromododecyloxy)­tetrahydro-2*H*-pyran (**14**)

3,4-Dihydropyran (0.76 g, 9.0 mmol), CH_2_Cl_2_ (75 mL), *p*-toluenesulfonic acid (*p*-TSA) (2 mg), and bromoalcohol **13** (1.92 g, 7.5 mmol) were stirred at room temperature for 8 h. The reaction was quenched by adding H_2_O (30 mL) and the aqueous media extracted with CH_2_Cl_2_. The organic layer was washed with saturated solution of NaHCO_3_, dried with Na_2_SO_4_, filtered, and concentrated under vacuum. The crude material was purified by flash chromatography (9.75:0.25 hexane/ethyl acetate), affording **14** in 95% yield.^19^ ^1^H NMR (400 MHz, CDCl_3_, ppm): δ 4.59 (t, 1H, *J* = 6.1 Hz), 3.84 (ddd, 2H, *J* = 11.0 Hz, *J* = 7.4 Hz, *J* = 3.2 Hz), 3.70 (dt, 2H, *J* = 9.4 Hz, *J* = 6.9 Hz), 3.47 (dt, 2H, *J* = 5.3 Hz, *J* = 4.8 Hz), 3.41–3.31 (m, 2H), 1.89–1.75 (m, 2H), 1.73–1.62 (m, 2H), 1.61 – 1.46 (m, 2H), 1.38 (dd, 2H, *J* = 14.0 Hz, *J* = 6.7 Hz), 1.34–1.18 (m, 16H). ^13^C NMR (100 MHz, CDCl_3_, ppm): δ 98.8, 67.6, 62.3, 34.0, 32.8, 30.8, 29.7, 29.5, 29.5, 29.5, 29.4, 29.4, 28.7, 28.1, 26.2, 25.5, 19.7. MS (EI, 70 eV; *m*/*z* (%)): 41 (15), 43 (11), 55 (23), 56 (20), 57 (15), 69 (14), 83 (10), 84 (15), 85 (100), 97 (3), 101 (3), 347 (1), 349 (1).

#### 2-(Tetradec-13-yn-1-yloxy)­tetrahydro-2*H*-pyran (**15**)

Lithium acetylide-ethylenediamine complex (79 mg; 0.855 mmol) and anhydrous DMSO (2.5 mL) were stirred at rt and cooled to 10 °C, followed by the slow addition of compound **14** (0.2 g, 0.57 mmol). The temperature was then raised to rt and the reaction stirred for 4 h. The reaction was quenched by the addition of cooled H_2_O (2 mL) and extracted twice with diethyl ether (5 mL). The organic phase was washed with H_2_O, dried with Na_2_SO_4_, and concentrated under reduced pressure. The crude product was purified by flash chromatography (8:2 hexane/ethyl acetate), affording **15**, in 97% yield.^20^ ^1^H NMR (400 MHz, CDCl_3_, ppm): δ 4.63–4.49 (m, 1H), 3.86 (ddd, 2H, *J* = 11.1 Hz, *J* = 7.4 Hz, *J* = 3.4 Hz), 3.72 (dt, 2H, *J* = 9.5 Hz, J = 6.9 Hz), 3.49 (dt, 2H, *J* = 5.0 Hz, J = 4.5 Hz), 3.37 (dt, 2H, *J* = 9.5, J = 6.7 Hz), 2.17 (td, 2H, *J* = 7.1 Hz, *J* = 2.6 Hz), 1.93 (t, 1H, *J* = 2.6 Hz), 1.82 (ddd, 2H, *J* = 14.9 Hz, *J* = 9.7 Hz, *J* = 3.6 Hz), 1.74–1.66 (m, 2H), 1.64–1.45 (m, 4H), 1.40–1.15 (m, 14H). ^13^C NMR (100 MHz, CDCl_3_, ppm): δ 98.8, 84.8, 68.0, 67.7, 62.3, 30.8, 29.7, 29.5, 29.4, 29.1, 28.7, 28.5, 26.2, 25.5, 19.7, 18.4. MS (EI, 70 eV; *m*/*z* (%)): 41 (19), 43 (11), 55 (23), 56 (18), 57 (10), 67 (20), 81 (15), 85 (100), 101 (32), 109 (3), 293 (1).

#### (*Z*)-2-(Octadec-15-en-13-yn-1-yloxy)­tetrahydro-2*H*-pyran (**19**)

(*Z*)-1-Iodo-1-butene (**18**) (0.87 g, 4.8 mmol), anhydrous THF (20 mL), piperidine (0.215 g, 2.5 mmol), Pd­(PPh_3_)_4_ (0.07 g, 0.06 mmol), and CuI (0.023 g, 0.12 mmol) were added to a round-bottom flask at rt under inert atmosphere. A solution of the alkyne **15** (0.700 g, 2.38 mmol) in THF (5 mL) was added dropwise to the reaction medium at rt. The resulting mixture was stirred for 5 h, followed by the addition of a saturated solution of NH_4_Cl and extraction with diethyl ether. The organic layer was washed with brine and dried with Na_2_SO_4_. After evaporating the solvent under vacuum, the crude product was purified by flash chromatography (9.5:0.5 hexane/diethyl ether), yielding **19** (87%).^21^ ^1^H NMR (400 MHz, CDCl_3_, ppm): δ 5.80 (dt, 1H, *J* = 10.7 Hz, J = 7.3 Hz), 5.40 (d, 1H, *J* = 10.7 Hz), 4.59–4.55 (m, 1H), 3.87 (ddd, 2H, *J* = 11.2, *J* = 7.4 Hz, *J* = 3.5 Hz), 3.73 (dt, 2H, *J* = 9.6 Hz, *J* = 6.9 Hz), 3.50 (ddd, 2H, *J* = 10.9 Hz, *J* = 5.2 Hz, *J* = 3.4 Hz), 3.38 (dt, 2H, *J* = 9.6 Hz, *J* = 6.7 Hz), 2.38–2.20 (m, 2H), 1.93–1.78 (m, 2H), 1.73–1.69 (m, 2H), 1.63–1.48 (m, 4H), 1.45–1.19 (m, 16H), 1.00 (t, 3H, *J* = 7.6 Hz). ^13^C NMR (100 MHz, CDCl_3_, ppm): δ 144.0, 108.7, 98.8, 94.5, 67.7, 62.3, 34.1, 30.8, 29.7, 29.6, 29.5, 29.5, 29.5, 29.1, 28.9, 26.2, 25.5, 23.4, 22.3, 19.7, 19.5, 14.0, 13.4. MS (EI, 70 eV; *m*/*z* (%)): 41 (13), 43 (9), 55 (16), 57 (9), 67 (17), 79 (20), 81 (9), 85 (100), 91 (11), 93 (12), 94 (19), 95 (12), 101 (11), 275 (3), 348 (1).

#### 2-((13*Z*,15*Z*)-Octadeca-13,15-dien-1-yloxy)­tetrahydro-2*H*-pyran (**20**)

Dicyclohexylborane was prepared by adding a solution of cyclohexene (0.846 g, 3.45 mmol) in diethyl ether (5 mL) to a borane–dimethyl sulfide complex solution (2.58 g, 5.13 mmol, 2 mol·L^–1^) at rt. A solution of **19** (0.600 g, 1.71 mmol) in dry diethyl ether (15 mL) was slowly added at 0 °C to the dicyclohexylborane solution, and the resulting mixture stirred for 4 h at rt. Glacial acetic acid (0.2 mL, 10.4 mmol) was added and the reaction stirred for 5 h at 60 °C. The reaction medium was treated with NaOH (0.8 mL, 6 mol·L^–1^ in H_2_O) followed by the slow addition of H_2_O_2_ (0.6 mL, 35%). The resulting mixture was stirred for 30 min, extracted with hexane, washed with brine, dried with Na_2_SO_4_, and the organic phase concentrated under vacuum. The crude product was purified by flash chromatography (9:1 hexane/diethyl ether), yielding **20** (75%).^20^ ^1^H NMR (400 MHz, CDCl_3_, ppm): δ 6.36–6.12 (m, 2H), 5.45 (dt, 1H, *J* = 10.2 Hz, *J* = 7.3 Hz), 5.42 (dt, 1H, *J* = 10.3 Hz, *J* = 7.2 Hz), 4.17–3.92 (m, 1H), 3.71–3.55 (m, 2H), 3.55 (dd, 2H, *J* = 12.2 Hz, *J* = 6.1 Hz), 3.50–3.34 (m, 4H), 2.30–2.06 (m, 2H), 1.81–1.50 (m, 8H), 1.44–1.14 (m, 16H), 0.99 (t, 3H, *J* = 7.5 Hz). ^13^C NMR (100 MHz, CDCl_3_, ppm): δ 133.5, 132.1, 123.5, 123.2, 98.8, 64.6, 62.3, 30.8, 30.3, 29.9, 29.9, 29.8, 29.7, 29.7, 29.6, 29.6, 29.6, 29.2, 27.4, 25.5, 20.7, 19.7, 14.2. MS (EI, 70 eV; *m*/*z* (%)): 41 (15), 43 (8), 55 (22), 67 (37), 68 (10), 69 (8), 79 (12), 81 (17), 82 (21), 83 (9), 85 (100), 95 (18), 96 (12), 101 (13), 266 (2), 332 (2), 350 (1).

#### (13*Z*,15*Z*)-Octadeca-13,15-dien-1-ol (**5**)

*p*-TSA crystals were added to a stirred solution of **20** (0.200 g, 0.57 mmol) in methanol (2 mL), and the mixture was stirred at rt for 5 h. The reaction was quenched by the addition of water, followed by extraction with diethyl ether and sequential washing with saturated NaHCO_3_ solution and brine. The organic layer was dried over anhydrous Na_2_SO_4_ and filtered. After solvent removal, the crude product was purified by flash chromatography using 8:2 hexane/ethyl acetate, affording compound **5** in 81% yield.^19^ ^1^H NMR (600 MHz, CDCl_3_): δ 6.27–6.19 (m, 2H), 5.44 (p, *J* = 7.2 Hz, 2H), 3.64 (t, *J* = 6.6 Hz, 2H), 2.21–2.13 (m, 4H), 1.56 (dt, *J* = 13.2, 6.7 Hz, 2H), 1.27 1.25 (m, 18H), 1.00 (t, *J* = 7.5 Hz, 3H). ^13^C NMR (151 MHz, CDCl_3_): δ 133.6, 132.2, 123.4, 123.0, 63.1, 32.8, 29.7, 29.6, 29.6, 29.6, 29.6, 29.5, 29.4, 29.3, 27.5, 25.7, 20.8, 14.2. MS (EI, 70 eV; *m*/*z* (%)): 41 (34), 43 (11), 53 (7), 54 (11), 55 (41), 67 (100), 68 (36), 69 (16), 77 (7), 79 (31), 80 (15), 81 (50), 82 (76), 83 (13), 93 (16), 94 (11), 95 (47), 96 (37), 97 (10), 109 (14), 110 (11), 121 (10), 266 (7).

#### (13*Z*,15*Z*)-Octadeca-13,15-dienal (**4**)

A mixture of pyridinium chlorochromate (PCC, 0.5 g), Celite (0.5 g), and sodium acetate (0.04 g) in dichloromethane (10 mL) was stirred at rt. A solution of alcohol **5** (0.2 g, 0.75 mmol) in dichloromethane (1 mL) was added dropwise to the stirred suspension, and the resulting mixture was stirred for 3 h. The reaction mixture was then eluted through a silica gel/Celite (1:1) column using diethyl ether, and the solvent was removed under reduced pressure. The crude product was purified by flash chromatography (8:2 hexane/ethyl acetate), affording compound **4** in 98% yield.^19^ ^1^H NMR (400 MHz, CDCl_3_): δ 9.76 (t, *J* = 1.9 Hz, 1H), 6.27–6.15 (m, 2H), 5.49–5.41 (m, 2H), 2.41 (td, *J* = 7.4, 1.9 Hz, 2H), 2.22–2.12 (m, 2H), 1.62 (dd, *J* = 7.6, 2.7 Hz, 2H), 1.26 (s, 18H), 1.00 (t, *J* = 7.6 Hz, 3H). ^13^C NMR (101 MHz, CDCl_3_): δ 202.9, 133.6, 132.2, 123.5, 123.1, 43.9, 29.7, 29.6, 29.6, 29.5, 29.4, 29.4, 29.3, 29.2, 27.5, 22.1, 20.8, 14.2. MS (EI, 70 eV; *m*/*z* (%)): 41 (32), 43 (9), 53 (7), 54 (11), 55 (33), 57 (6), 67 (100), 68 (37), 69 (13), 79 (20), 80 (10), 81 (47), 82 (67), 83 (11), 93 (10), 94 (7), 95 (47), 96 (27), 97 (8), 98 (8), 109 (13), 110 (9), 264 (10).

#### (13*Z*,15*Z*)-Octadeca-13,15-dien-1-yl acetate (**9**)

A solution containing alcohol **5** (0.200 g, 0.75 mmol), pyridine (0.181 mL), and acetic anhydride (0.212 mL) in dichloromethane (10 mL) was stirred overnight at rt. The resulting mixture was diluted with diethyl ether, washed successively with water, saturated NaHCO_3_ solution, and brine. The organic phase was dried over anhydrous Na_2_SO_4_ and concentrated under reduced pressure. The crude product was purified by flash chromatography (9:1 hexane/diethyl ether), affording **9** in 95% yield.^22^ ^1^H NMR (400 MHz, CDCl_3_): δ 6.32–6.14 (m, 2H), 5.53–5.38 (m, 2H), 4.05 (t, *J* = 6.8 Hz, 2H), 2.25–2.08 (m, 4H), 2.04 (s, 3H), 1.61 (t, *J* = 7.1 Hz, 2H), 1.26 (bs, 18H),1.00 (t, *J* = 7.6 Hz, 3H). ^13^C NMR (101 MHz, CDCl_3_): δ 171.2, 133.6, 132.2, 130.9, 128.9, 123.5, 123.1, 64.7, 29.7, 29.6, 29.6, 29.5, 29.3, 29.3, 28.6, 27.5, 25.9, 21.0, 20.8, 14.2. MS (EI, 70 eV); *m*/*z* (%)): 41 (32), 43 (53), 53 (7), 54 (10), 55 (42), 57 (8), 61 (7), 67 (100), 68 (32), 69 (19), 79 (51), 80 (28), 81 (53), 82 (67), 83 (16), 93 (28), 94 (24), 95 (52), 96 (45), 97 (12), 107 (13), 108 (12), 109 (18), 110 (17), 121 (23), 122 (10), 123 (10), 124 (10), 135 (16), 149 (7), 248 (7), 308 (12).

#### (*Z*)-1-Iodobut-1-ene (**18**)

1-Butyne (**16**, 3.0 mL, 37 mmol) was added to diethyl ether (100 mL) at 0 °C and the mixture was cooled to −70 °C. A solution of MeLi in diethyl ether (34 mL, 55.5 mmol, 1.6 mol·L^–1^) was then added and the resulting mixture stirred at −50 °C for 1 h. The solution was cooled to −70 °C and a solution of iodine (14.0 g, 55.5 mmol) in diethyl ether (150 mL) was added. The mixture was then stirred at −70 °C for 1 h and rt overnight. Water was then added and the aqueous medium extracted with diethyl ether. The organic layer was washed with an aqueous solution of NaHSO_3_ (10%) and brine, and dried with Na_2_SO_4_. The crude product was distilled (100 mbar, 80 °C), yielding 1-iodobut-1-yne (**17**). Dicyclohexylborane was prepared by adding a solution of cyclohexene (4.1 g, 49.6 mmol) in diethyl ether (5 mL) to a borane dimethyl sulfide complex solution (12.4 mL, 24.8 mmol, 2 mol·L^–1^) at rt. A solution of 1-iodobut-1-yne (**17**, 3 g, 16.6 mmol) in dry diethyl ether (100 mL) was slowly added at 0 °C to the dicyclohexylborane solution. The mixture was then stirred for 1 h, heated to rt and stirred for 3 h more. Glacial acetic acid (4 mL, 83 mmol) was added and the reaction stirred overnight at rt. Diethyl ether was added and the aqueous phase neutralized with NaHCO_3_ saturated solution. The mixture was extracted with diethyl ether and the organic phase concentrated under vacuum. The crude product was distilled (100 mbar/75 °C), to furnish **18** in 38% yield over the two steps.^23^ ^1^H NMR (400 MHz, CDCl_3_, ppm): δ 6.31–5.93 (m, 2H), 2.24–1.97 (m, 2H), 1.13 (t, 3H, *J* = 6.6 Hz). ^13^C NMR (100 MHz, CDCl_3_, ppm): δ 142.72, 81.46, 28.25, 12.40.

### Field Bioassays

Field experiments were carried out in a yerba mate plantation in São Mateus do Sul, Paraná, Brazil (25°54′57”S 50°17′24”W). Rubber septa (10 mm O.D. × 18 mm) (Aldrich Chemical Co., USA) were used as dispensers. Three treatments, A, B, and C, were tested in triplicate. In treatment A, 1 mg of each compound **4**, **5**, and **9** was applied; in B, 1 mg of compound **9** alone; and C served as control with no pheromonal components. Compounds were dissolved in 150 μL of hexane and loaded onto the septa. The baited septa were placed in “Delta” traps (Biocontrole, Brazil) at 1.5 m above ground, attached to yerba mate branches. Traps were arranged in a randomized block design, spaced 150 m apart, and left in the field for 14 days, with inspections every 48 h. Captured insects were counted, and the mean captures and standard deviations were calculated for each treatment. Data were statistically analyzed by ANOVA followed by Tukey’s HSD test.^24^

## Results and Discussion

GC-EAD analyses, in which antennae of male *T. camina* were subjected to volatiles derived from pheromonal gland extracts of virgin females, consistently yielded nine distinct electrophysiological responses (**1**–**9**, Figure 1). These signals were associated with compounds exhibiting the following retention indexes on a DB-5 column: 2014 (**1**), 2064 (**2**), 2072 (**3**), 2081 (**4**), 2143 (**5**), 2202 (**6**), 2250 (**7**), 2258 (**8**), and 2269 (**9**).

**Figure 1:**
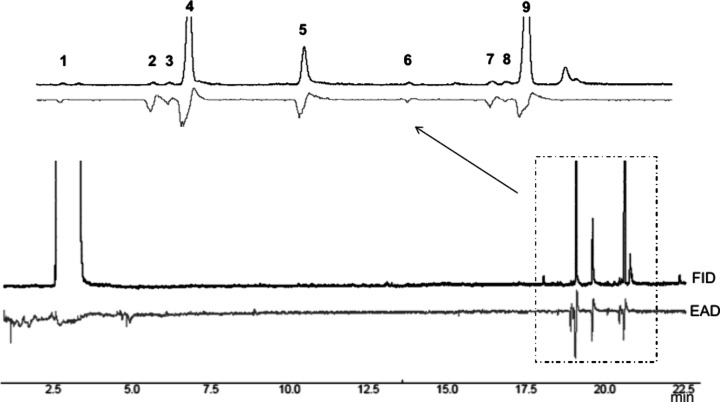
Gas chromatography coupled with electroantennographic detection (GC–EAD) analysis using a *Thelosia camina* male antenna in response to extracts from virgin female glands (lower trace) vs the GC-FID trace. Numbers above the peaks indicate electrophysiological responses corresponding to compounds **1**–**9**. The dotted square indicates the zoomed-in region where the responses are highlighted.

All nine identified compounds exhibited fragmentation profiles characteristic of Type I lepidopteran pheromones, as exemplified in Figure 2. For clarity, only the spectra of major compounds **4**, **5**, and **9** are shown and discussed in detail. The mass spectrum of compound **4** (Figure 2, MS 4) displays a molecular ion at *m*/*z* 264, consistent with a molecular composition C_18_H_3__2_O. A prominent fragment at *m*/*z* 235 (M^+^ – 29), corresponding to the loss of CHO indicates the presence of an aldehyde group within the carbon chain, while the degree of unsaturation supports two additional double bonds. Compounds **2** and **3** exhibited nearly identical fragmentation patterns (Supporting Information), indicating that compounds **2**, **3**, and **4** are stereoisomeric aldehydes.

**Figure 2:**
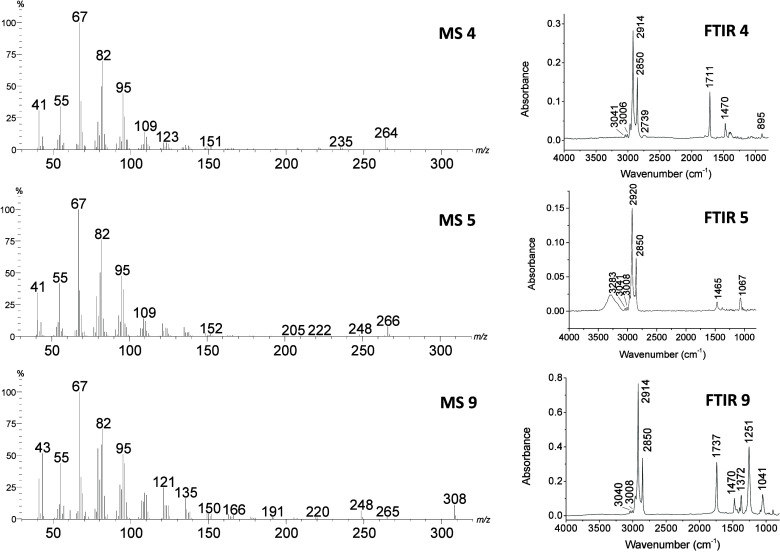
Mass (MS) and infrared (IR) spectra of the major pheromone components **4** (aldehyde), **5** (alcohol), and **9** (acetate) isolated from female *T. camina* pheromone glands.

Compound **5** (Figure 2, MS 5) shows a molecular ion at *m*/*z* 266, consistent with a molecular composition C_18_H_3__4_O, and a fragment at *m*/*z* 248 (M^+^ – 18) corresponding to the loss of H_2_O, suggesting the presence of a hydroxyl group. Its degree of unsaturation likewise supports two C–C double bonds. The mass spectrum of compound **9** (Figure 2, MS 9) exhibits a molecular ion at *m*/*z* 308 suggesting a molecular composition C_20_H_36_O_2_ with three unsaturations. The fragment at *m*/*z* 248 (M^+^ – 60), corresponding to the loss of acetic acid, together with a strong ion at *m*/*z* 43, are diagnostic of an acetate ester. Compounds **7** and **8** showed highly similar fragmentation profiles to that of compound **9**, indicating that they likely represent stereoisomeric or positional variants of the same acetate backbone (see Supporting Information).

The functional groups inferred from MS for compounds **4**, **5**, and **9** were corroborated by FTIR analysis. Compound **4** showed a C=O stretch at 1711 cm^–1^ and aldehyde C–H band at 2739 cm^–1^ (Figure 2, IR4); compound **5** presented a broad O–H stretch at 3283 cm^–1^ (Figure 2, IR5); and compound **9** exhibited both a C=O stretch at 1737 cm^–1^ and a C–O stretching band at 1251 cm^–1^, consistent with the presence of an ester group (Figure 2, IR9). The retention index (RI) differences among the three natural components aligned with the expected RI offset typically observed for Type I Lepidopteran aldehydes, alcohols (increase of ∼60 units), and acetates (increase of ∼125 units),^25^ further supporting their structural assignments.

The relatively high intensity of the molecular ion (M^+^) in compounds **4**, **5**, and **9** suggests the presence of a conjugated diene system.^26^ Moreover, the mass spectrum of these three compounds suggests the presence of a conjugated ω3,ω5-diene system, characterized by fragment ions at *m*/*z* 67, 79, 81, 82, and 95 (compound **4**); *m*/*z* 67, 81, 82, and 95 (compound **5**); and *m*/*z* 67, 81, 82, and 95 (compound **9**).^27^ This hypothesis was confirmed by derivatizing the natural extract with the dienophile 4-methyl-1,2,4-triazoline-3,5-dione (MTAD)^17,18^ and analyzing the products by GC–MS. Two addition adducts were detected—compounds **10** and **11**—which were likely originated from two conjugated dienyl structures containing an acetate and an aldehyde group, respectively (Figure 3A).

**Figure 3:**
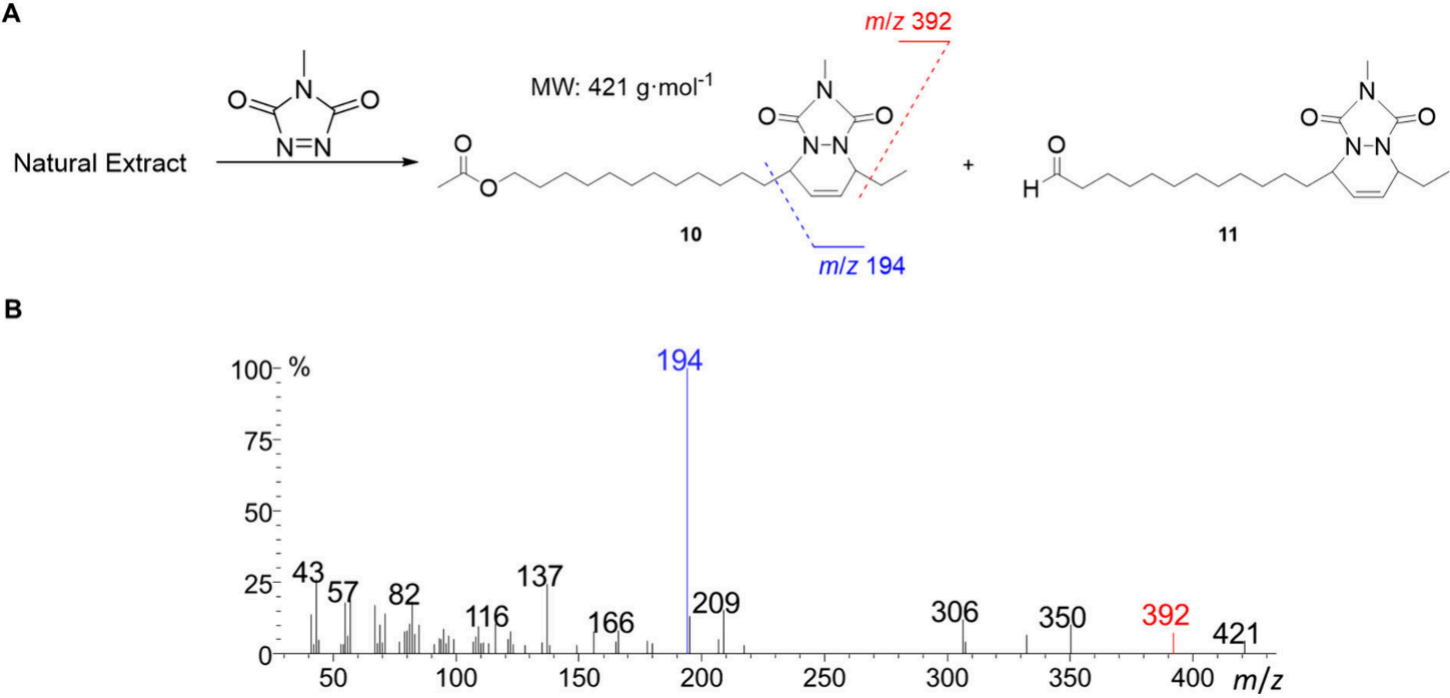
(A) Derivatization of the natural pheromone extract of *T. camina* with MTAD results in the formation of two addition adducts, compounds **10** and **11**, corresponding to the reaction of the conjugated dienes of acetate **9** and aldehyde **4**, respectively. (B) Mass spectrum of compound **10**, the acetate-containing MTAD adduct, highlighting diagnostic fragment ions (*m*/*z* 194 and 392) that confirm the positions of the double bonds in the parental diene.

The mass spectrum of derivative **10** (Figure 3B) displays two relatively intense fragments at *m*/*z* 194 and 392, corresponding to cleavages adjacent to the six-membered ring. These fragments reveal the positions of the double bonds in the parental diene, along with a molecular ion peak at *m*/*z* 421. The mass spectrum of compound **11** (Figure S2, Supporting Information) showed similarly intense fragmentations at the same positions along the chain (*m*/*z* 194 and 377), suggesting that the natural dienes originally contained C–C double bonds at positions 13 and 15.

Infrared spectroscopy provided key information to determine the stereochemistry of the double bonds in natural compounds **1**–**9**. As representative examples, comparison of infrared spectra (GC-FTIR) of compounds **7**, **8**, and **9** revealed typical signatures of conjugated dienes, in addition to the characteristic bands of the acetate group (C–O stretching at 1250 cm^–1^ and C=O stretching at 1741 cm^–1^). In the case of compound **9**, similarly as for compounds **4** and **5** (Figure 4, IR 9, Expansion A), the spectrum displayed the typical pattern for *Z*,*Z*-conjugated dienes, with bands at 3040 and 3008 cm^–1^ corresponding to the axial stretching of =C–H. For compounds **7** and **8,** similarly as for compounds **2** and **3,** respectively, these bands appeared at 3023 and 3008 cm^–1^, along with out-of-plane angular deformation bands at 951 and 986 cm^–1^, which are considered fingerprint signals for *E*,*Z*- or *Z*,*E*-conjugated double bonds (Figure 4, IR 7 and IR 8, Expansion B).^28^ Under the DB-5 column conditions used, the elution order of conjugated dienes is well established in the literature as *Z*,*E* eluting slightly before than *E*,*Z*, followed by *Z*,*Z* and finally *E*,*E*).^29^ Since the minor dienes **7** and **8** (and likewise **2** and **3**) eluted immediately before the major *Z*,*Z* isomers **9** and **4**, their retention time clearly confirms them as *Z*,*E* and *E*,*Z* isomers, respectively (see Figure 1).

**Figure 4:**
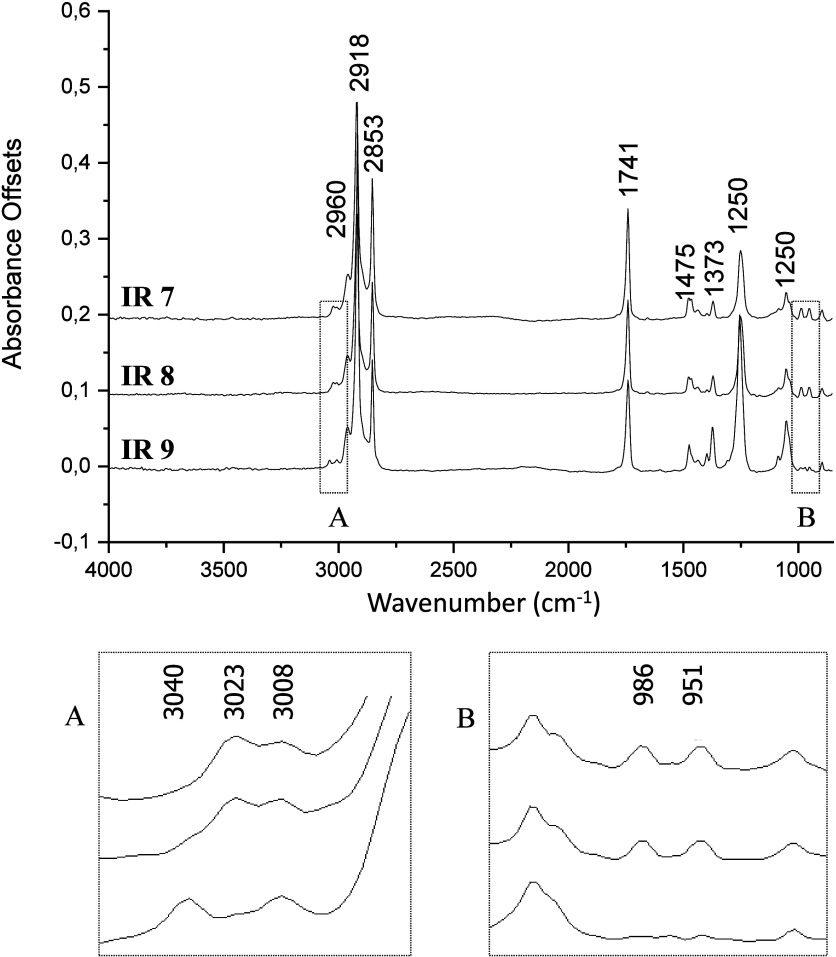
Representative GC-FTIR spectra of compounds **7**, **8**, and **9** isolated from female *T. camina* pheromone glands, highlighting diagnostic absorption bands for conjugated dienes and acetates. Expansion A shows the *Z*,*Z*-conjugated diene pattern of compound **9**, while Expansion B illustrates the *E*,*Z*/*Z*,*E* signatures observed in compounds **7** and **8**.

To finalize the full identification of all nine pheromone components, the minor constituents **1** and **6** were unambiguously assigned as the monoenes *Z*13–C18:Ald and *Z*13–C18:Ac, respectively, based on their diagnostic fragmentation patterns (see Supporting Information) and retention indexes. These identifications were corroborated by coinjection with authentic synthetic standards.

The combined chromatographic and spectroscopic data support the structural proposal of the natural compounds as follows: (*Z*)-octadec-13-enal (**1**), (13*Z*,15*E*)-octadeca-13,15-dienal (**2**), (13*E*,15*Z*)-octadeca-13,15-dienal (**3**), (13*Z*,15*Z*)-octadeca-13,15-dienal (**4**), (13*Z*,15*Z*)-octadeca-13,15-dien-1-ol (**5**), (13*Z*)-octadec-13-en-1-yl acetate (**6**), (13*Z*,15*E*)-octadeca-13,15-dien-1-yl acetate (**7**), (13*E*,15*Z*)-octadeca-13,15-dien-1-yl acetate (**8**), and (13*Z*,15*Z*)-octadeca-13,15-dien-1-yl acetate (**9**) (Figure 5).

**Figure 5:**
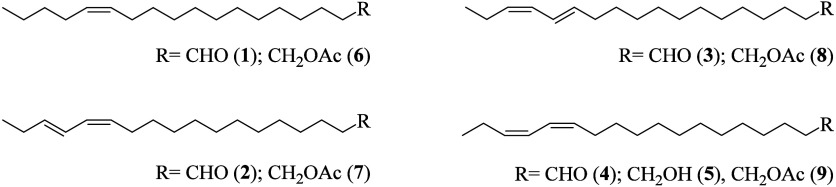
Proposed structures for the nine GD-EAD active compounds **1**–**9**: (*Z*)-octadec-13-enal (**1**), (13*Z*,15*E*)-octadeca-13,15-dienal (**2**), (13*E*,15*Z*)-octadeca-13,15-dienal (**3**), (13*Z*,15*Z*)-octadeca-13,15-dienal (**4**), (13*Z*,15*Z*)-octadeca-13,15-dien-1-ol (**5**), (13*Z*)-octadec-13-en-1-yl acetate (**6**), (13*Z*,15*E*)-octadeca-13,15-dien-1-yl acetate (**7**), (13*E*,15*Z*)-octadeca-13,15-dien-1-yl acetate (**8**), (13*Z*,15*Z*)-octadeca-13,15-dien-1-yl acetate (**9**).

The three major natural compounds—**4**, **5**, and **9**, all exhibiting *Z*,*Z* double bond stereochemistry—were synthesized via a linear approach, with key steps involving lithium anion-mediated coupling and Sonogashira cross-coupling reactions (Scheme 1). The synthesis began with the monobromination of 1,12-dodecanediol (**12**) with HBr, followed by protection of the resulting bromoalcohol (**13**) as the corresponding THP ether, affording compound **14** in 84% overall yield for the two steps. Subsequently, compound **14** was treated with lithium acetylide–ethylenediamine complex in DMSO, furnishing compound **15** in excellent yield.

**Scheme 1:**
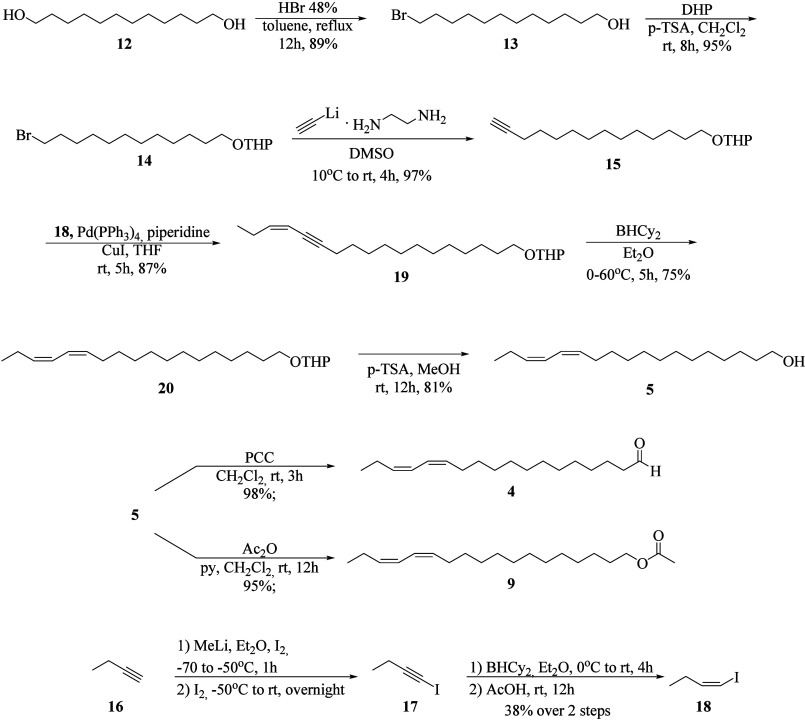
Synthetic Strategy Employed for the Preparation of the Three Major Pheromone Components **4**, **5**, and **9**, All Bearing *Z*,*Z*-Configured Conjugated Dienes

To enable a cross coupling reaction with alkyne **15**, which already contains a triple bond at position 13, the vinyl iodide **18** was prepared via lithiation of 1-butyne (**16**), followed by quenching with iodine and selective *Z*-reduction of the triple bond using dicyclohexylborane. The Sonogashira coupling between alkyne **15** and iodide **18** afforded the corresponding enyne **19** protected as THP ether. Subsequent *Z*-reduction of the triple bond in **19** with dicyclohexylborane, followed by deprotection of the hydroxyl group, yielded (13*Z*,15*Z*)-octadeca-13,15-dien-1-ol (**5**). Oxidation of alcohol **5** with PCC furnished (13*Z*,15*Z*)-octadeca-13,15-dienal (**4**), while acetylation provided (13*Z*,15*Z*)-octadeca-13,15-dien-1-yl acetate (**9**) in good yields.

The mass and infrared spectra, along with the RIs of the three major components **4**, **5**, and **9**, matched those of the synthesized standards, confirming the initially proposed structures. Similarly, the minor compounds (13*E*,15*Z*)-octadeca-13,15-dienal (**3**) and (13*E*,15*Z*)-octadeca-13,15-dien-1-yl acetate (**8**) were prepared in three steps from intermediate **19** by selective triple-bond reduction to the *E* isomer using LiAlH_4_. The synthetic routes were carried out with the purpose of generating standards to confirm the geometry of the minor dienes. Structural identity was established exclusively by comparison of their retention times, mass spectra, and infrared profiles with those of the natural extract, all of which were in full agreement.

Preliminary field assays evaluated the attractiveness of the identified pheromone components by determining the mean number of male *T. camina* captured per treatment. Traps baited with either the major acetate, 13*Z*,15*Z*-18Ac (**9**), or the ternary blend of major compounds—13Z,15*Z*-18Ald (**4**), 13*Z*,15*Z*-18OH (**5**), and 13*Z*,15*Z*-18Ac (**9**) in 1:1:1 ratio—captured significantly more insects than control traps, demonstrating a strong attractant activity under natural conditions. No significant difference was observed between traps containing only compound **9** and those with the ternary mixture, indicating that the acetate alone is sufficient to elicit a robust male response (Figure 6). The minor dienes and monoenes identified were not included in these initial assays due to limited synthetic availability and, since they could be considered byproducts of biosynthetic origin, were not expected to substantially affect attraction. However, minor components could exert synergistic effects enhancing the overall attractiveness of the lures as cited below.

**Figure 6:**
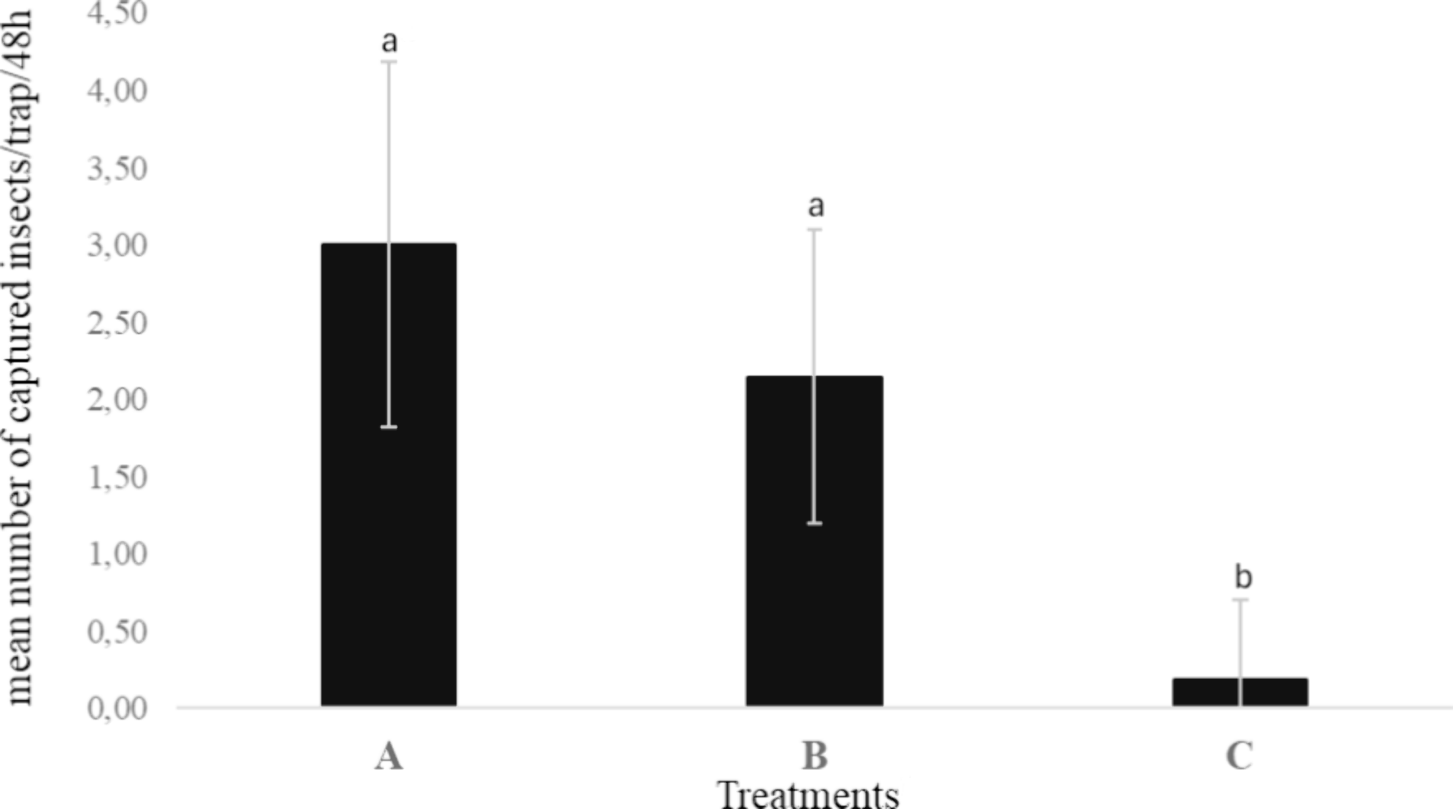
Mean numbers of insects captured in a field experiment under different treatments, for a period of 14 days. Treatments: A, mixture of compounds **4**, **5**, and **9** in 1:1:1 ratio; B, compound **9** alone; C, control. Different letters a and b indicate statistical differences by ANOVA followed by Tukey HSD test (*p* < 0.01).

Although these preliminary results provide clear evidence for the biological relevance of the major components, further field studies are required to optimize operational parameters such as dose, release rate, blend ratios including different mixtures of all compounds **1**–**9** to disclose any potential synergistic effect, and trap design. Altogether, these findings highlight the practical potential of the major pheromone compounds and the need for more comprehensive experiments to refine and maximize lure performance for monitoring and management of *T. camina* populations in yerba mate crops.

This study shows that the sex pheromone components of *T. camina* are consistent with the Type I category while presenting an uncommon predominance of C18-chain molecules. Most known Type I pheromones across Lepidoptera rely on shorter backbones with carbon chain lengths between 12 and 16 as documented by Ando et al.^30^ and Witzgall et al.^31^ The compounds identified from *Thelosia camina,* (13*Z*,15*Z*)-octadeca-13,15-dienal (**4**), (13*Z*,15*Z*)-octadeca-13,15-dien-1-ol (**5**), and (13*Z*,15*Z*)-octadeca-13,15-dien-1-yl acetate (**9**) represent, to the best of our knowledge, novel chemical structures in the context of insect pheromones. Moreover, this is the first report of a pheromone identified within the family Apatelodidae, which belongs to the superfamily Bombycoidea. This discovery considerably expands the chemical diversity known for Lepidopteran pheromones, particularly within this basal lineage of bombycoids.

In other members of Bombycoidea, such as *Samia cynthia* (*S. cynthia*; Saturniidae) and *Bombyx mori* (*B. mori*; Bombycidae), the sex pheromones are also conjugated dienes derived from long-chain fatty acids, but typically possess C16 backbones and an (*E*,*Z*)-configuration of the double bonds.^32,33^ In *B. mori*, for instance, Bjostad and Roelofs (1984)^34^ demonstrated that monounsaturated fatty acyl precursors such as (*Z*)-11-hexadecenoate can be further desaturated to yield conjugated (10*E*,12*Z*)-hexadecadienoate intermediates, which are then converted to the biologically active alcohols and aldehydes. This pathway provides a conceptual model of how conjugated dienes can arise through sequential desaturation and subsequent functional modification of fatty acid precursors. The conjugated (13*Z*,15*Z*)-octadecadienyl system in *T. camina*, with its longer chain and unique double-bond geometry, likely arises from stearic acid. While a loose analogy with *B. mori* can be drawn regarding conjugated diene formation, this remains speculative, and future studies are required to elucidate the enzymatic mechanism responsible for the *Z*,*Z*-18-carbon system, which differs from those described in other Bombycoidea lineages.

In summary, the sex pheromone of *Thelosia camina* has been successfully identified and synthesized, and its biological activity validated through field assays. This discovery lays the foundation for the development of pheromone-based tools for monitoring and managing this species, offering a promising avenue for sustainable pest control strategies.

## Supplementary Material



## Notes

Additional experimental procedures, MS spectra of all natural compounds, and ^1^H and ^13^C NMR spectra of synthetic compounds (PDF)

## #

Coordination of Agroindustry, Instituto Federal do Espírito Santo, Av. Elizabeth Minete, 500, Venda Nova do Imigrante–ES, 29.375-000, Brazil
