# Supplementary material for: Uncommon C18 Conjugated Dienes Define the Sex Pheromone System of Thelosia camina (Lepidoptera: Apatelodidae), a Pest of Yerba Mate
Source: J Agric Food Chem. 2026 Jan 4;74(1):569–78. doi: 10.1021/acs.jafc.5c14499 (PMC12814357; doi:10.1021/acs.jafc.5c14499)
Supplement: Supplementary file 1 [file jf5c14499_si_001.pdf]

SUPPORTING INFORMATION FOR

Journal of Agricultural and Food Chemistry

Uncommon C18 Conjugated Dienes Define the Sex Pheromone System of *Thelosia camina* (Lepidoptera: Apatelodidae), a Pest of Yerba Mate

Diogo M. Vidal[a,b], Emir B. Saad[b], Miryan D. A. Coracini[c], Rafael C. G. Pereira[a,d], Marcílio J. Thomazini[e], Carme Quero[f], Maria P. Bosch[f], Ángel Guerrero[f], Paulo H. G. Zarbin[b*]

[a] *Department of Chemistry, Universidade Federal de Minas Gerais, Av. Antônio Carlos, 6627, Belo Horizonte-MG, 31.270-901, Brazil*

[b] *Department of Chemistry, Universidade Federal do Paraná, Av. Cel. Francisco H. dos Santos, 100, Curitiba-PR, 81.531-990, Brazil*

[c] *Biological Sciences and Health Center, UNIOESTE, Rua Universitária, 1619, Cascavel-PR, 85.819-110, Brazil*

[d] *Coordination of Agroindustry, Instituto Federal do Espírito Santo, Av. Elizabeth Minete, 500, Venda Nova do Imigrante-ES, 29.375-000, Brazil*

[e] *Embrapa Florestas, BR-476, km17, s/n, Colombo-PR, 83.411-000 Brazil*

[f] *Department of Biological Chemistry, Institute of Advanced Chemistry of Catalunya-CSIC, Jordi Girona,18-26, Barcelona, 08034, Spain*

*Email: pzarbin@ufpr.br; Phone: (55) 41 33613174

CONTENTS

- 1. SUPPORTING FIGURES AND SCHEMES: pages 3-6

- 2. MS SPECTRA OF NATURAL COMPOUNDS **1-9**, OBTAINEDBY GC-MS: pages 7-9

- 3. EXPERIMENTAL PROCEDURES pages 10-12

- 3. NMR SPECTRA OF SYNTHETIC COMPOUNDS: pages 13-29

1. SUPPORTING FIGURES AND SCHEMES

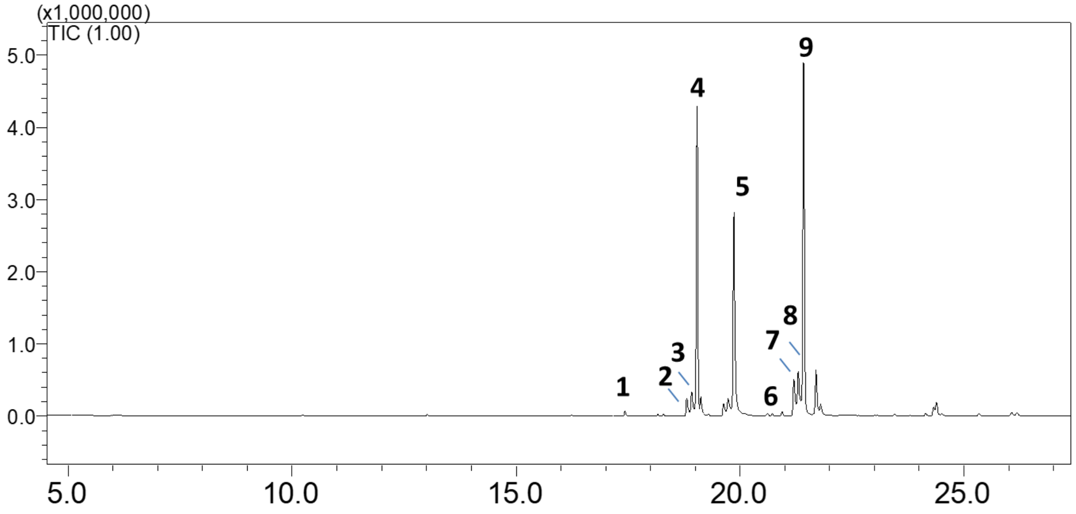

Figure S1: GC–MS chromatogram of extracts from the pheromone glands of female *Thelosia camina* adults.

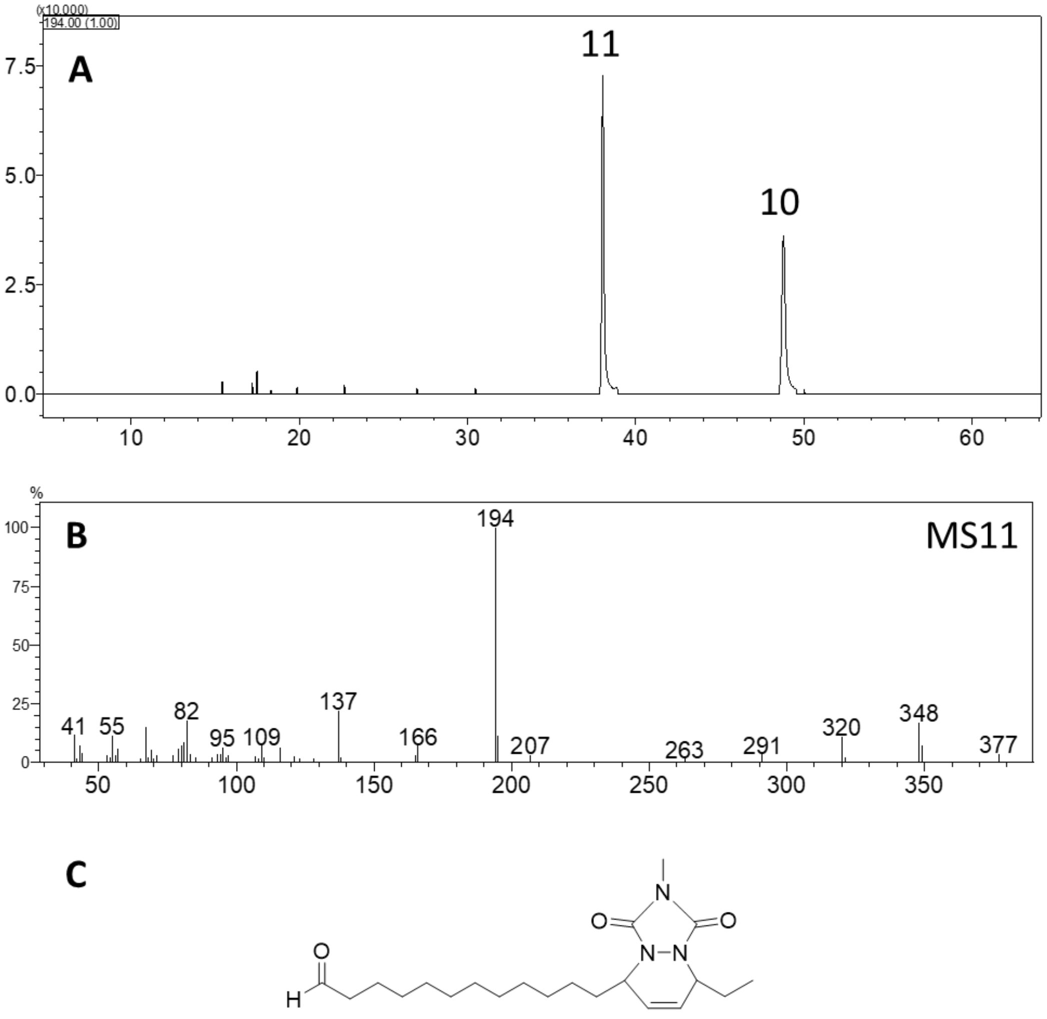

11

Molecular Weight: 377,53

Figure S2: (**A**) SIM (*m/z* 194) chromatogram obtained by GC-MS, showing peaks corresponding to compounds **10** (MS spectrum in Figure 3B, main text) and **11**. (**B**) Mass spectrum of the MTAD derivative **11**. (C) Chemical structure of **11**.

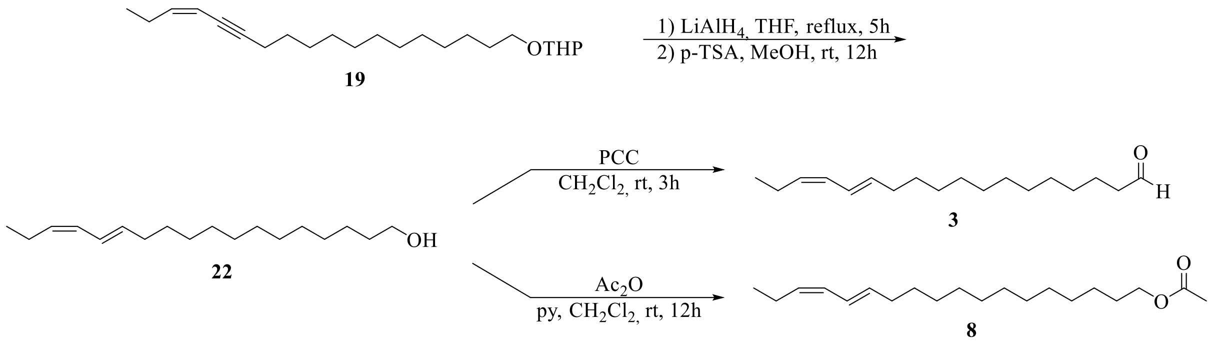

Scheme S1: Synthetic strategy employed for the microscale preparation of **21**, **3**, and **8**, all bearing 13*E*,15*Z*-configured conjugated dienes.

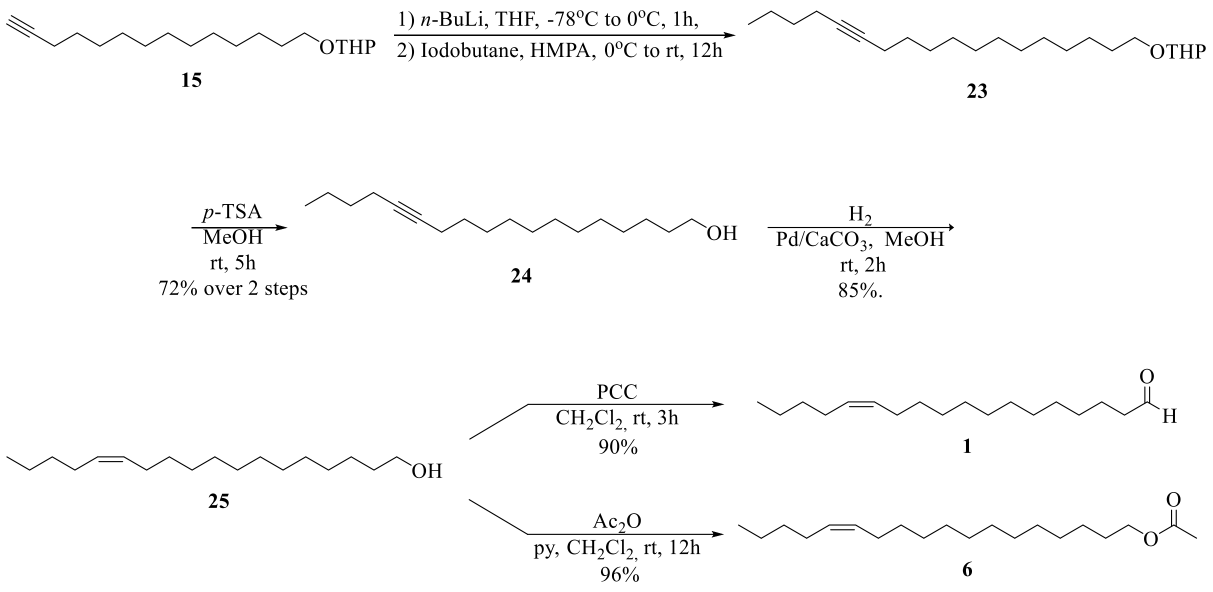

Scheme S2: Synthetic strategy employed for the preparation of the monoenes **1** and **6**.

2. MS SPECTRA OF NATURAL COMPOUNDS **1-9**, OBTAINED BY GC-MS

2.1 Compound **1**

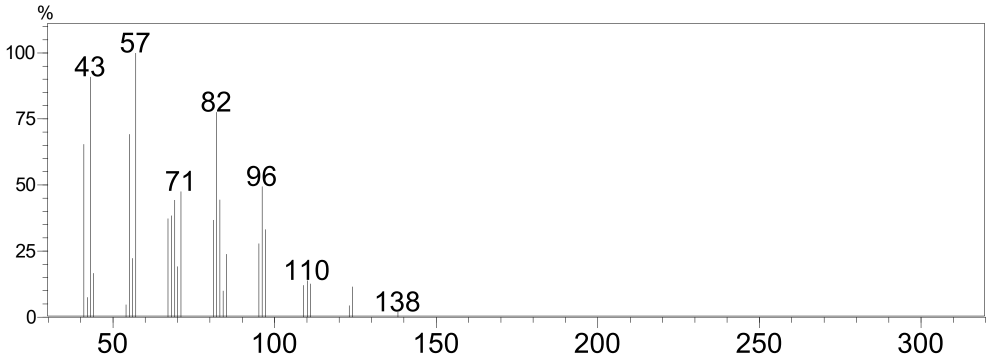

2.2 Compound **2**

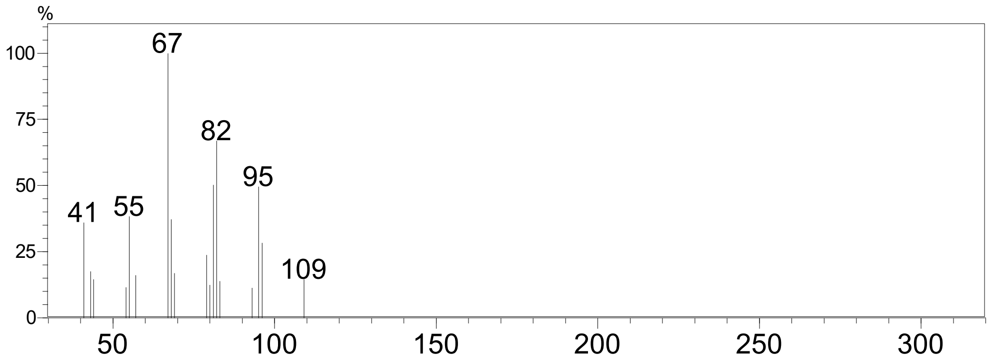

2.3 Compound **3**

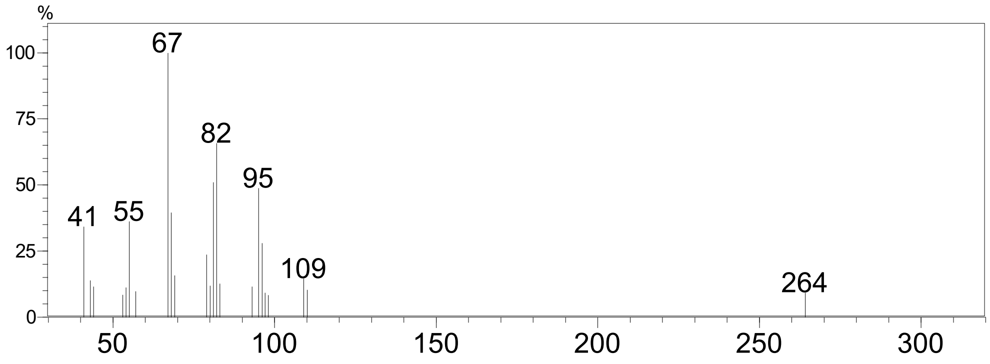

2.4 Compound **4**

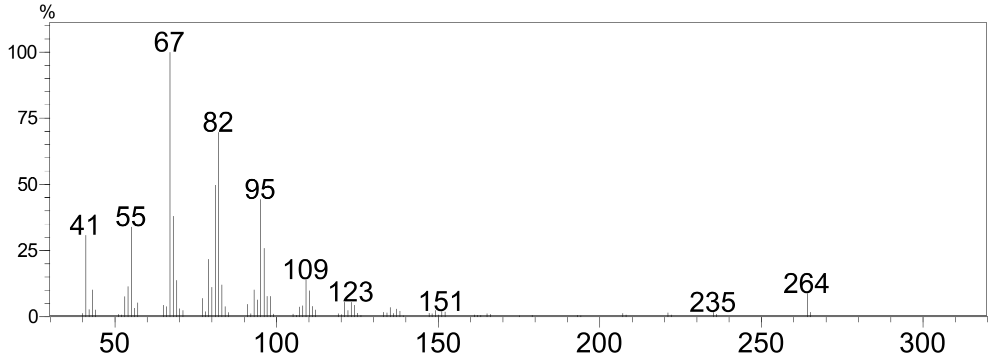

2.5 Compound **5**

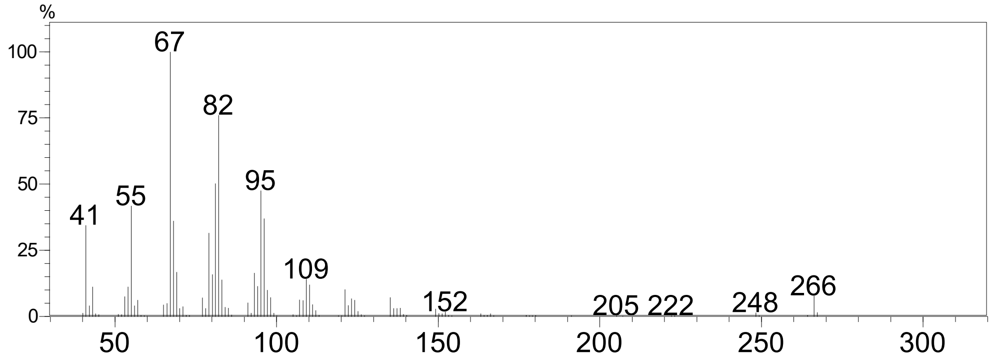

2.6 Compound **6**

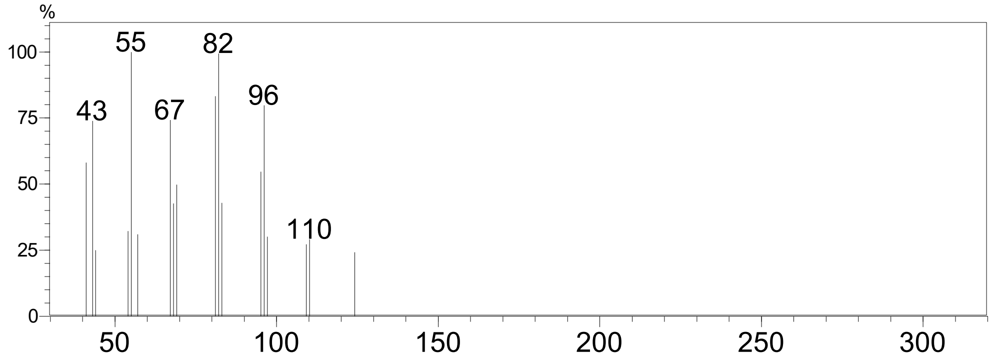

2.7 Compound **7**

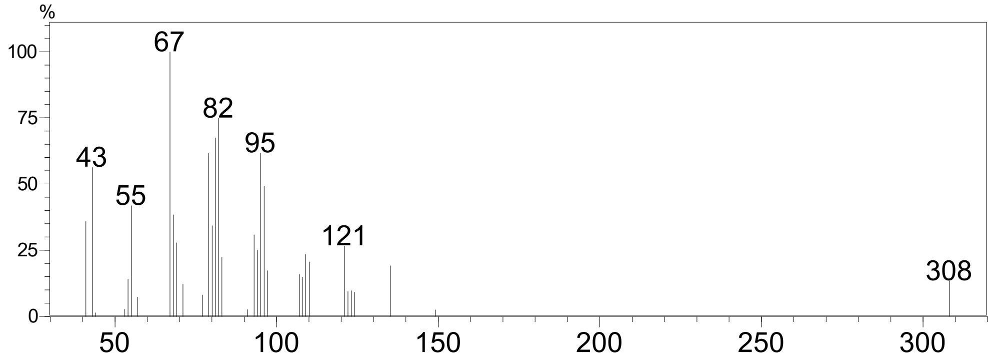

2.8 Compound **8**

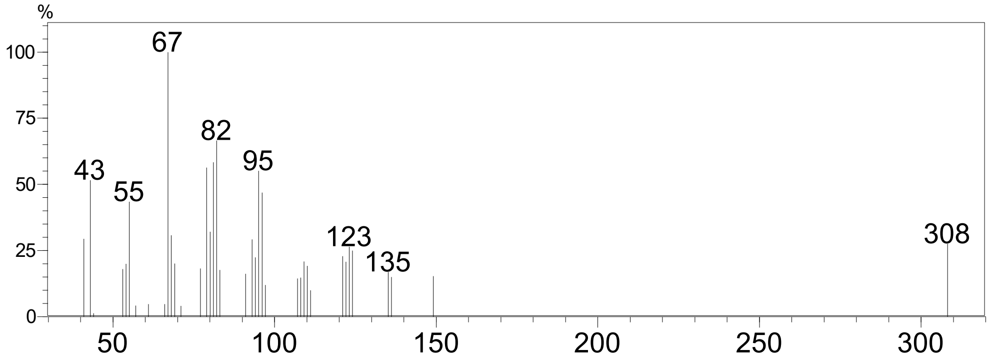

2.9 Compound **9**

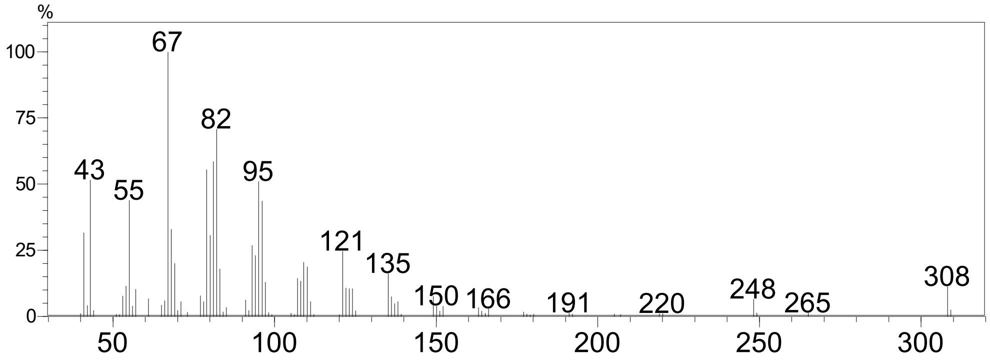

3. EXPERIMENTAL PROCEDURES

*2-((13E,15Z)-octadeca-13,15-dien-1-yloxy)tetrahydro-2H-pyran (**21**).* A suspension of $LiAlH_4$ (28 mg, 76 μmol) in anhydrous THF (300 μL) was prepared in an ampoule, maintained at room temperature under inert atmosphere and magnetic stirring. Compound **19** (22 mg, 76 μmol, in 200 μL of THF) was slowly added, the ampoule sealed, and the resulting mixture heated to 70 °C for 5 hours. After this period, the reaction was quenched by slow addition of cold water (100 μL), followed by aqueous NaOH (15%, 50 μL), and a second portion of cold water (100 μL). The resulting suspension was filtered, and the phases separated. The organic layer was dried over anhydrous Na_2SO_4 and concentrated under reduced pressure. The crude product was filtered through a Pasteur pipette filled with silica gel, eluting with hexane/ethyl acetate (9:1), to afford a mixture of the isomers 13*E*,15*Z* (**21**) and 13*Z*,15*Z* (**20**) in a 3:1 ratio.[1] MS (EI, 70eV): *m/z* (%): 41 (15), 43 (10), 55 (22), 67 (37), 68 (10), 69 (8), 79 (12), 81 (16), 82 (21), 83 (9), 85 (100), 95 (18), 96 (14), 101 (13), 266 (2), 332 (2), 350 (1).

*(13E,15Z)-octadeca-13,15-dien-1-ol (**22**).* This compound was produced in microscale following a method analogous to that used to prepare compound **5**, starting from **21**.

*(13E,15Z)-octadeca-13,15-dienal (**3**).* This compound was produced in microscale following a method analogous to that used to prepare compound **4**, starting from **22**.

*(13E,15Z)-octadeca-13,15-dien-1-yl acetate (**8**).* This compound was produced in microscale following a method analogous to that used to prepare compound **9**, starting from **22**.

*Octadec-13-yn-1-ol (**24**).* A solution of compound **15** (0.2 g, 0.68 mmol) in THF (2.0 mL) was prepared under inert atmosphere and cooled to –78 °C. *n*-BuLi (0.560 mL, 0.88

mmol, 1.57 M) was added dropwise, followed by gradual warming to 0 °C and stirring for 30 min. A solution of 1-iodobutane (0.162 g, 0.88 mmol) in anhydrous HMPA (0.2 mL) was then added, and the mixture stirred at 0 °C for 8 h. The reaction was diluted with hexane, and the organic layer was washed with distilled water and saturated NaCl solution, dried over anhydrous Na_2SO_4, and concentrated under reduced pressure, yielding 2-(octadec-13-yn-1-yloxy)tetrahydro-2H-pyran (**23**), which was used in the next step without further purification.

Compound **23** (100 mg, 0.285 mmol) was dissolved in methanol (1.0 mL), and a few crystals of *p*-TSA were added. The mixture was stirred at rt for 5 h, then quenched with water and extracted with diethyl ether. The organic layer was washed with saturated $NaHCO_3$ solution, dried over anhydrous Na_2SO_4, and concentrated under reduced pressure. The crude product was purified by flash chromatography (hexane/ethyl acetate 8:2), affording compound **24** in 72% yield over the two steps. [1, 2] ^{1}H NMR (400 MHz, $CDCl_3$, ppm) δ: 3.63 (t, 2H, *J* = 6.6 Hz), 2.32 – 2.02 (m, 4H), 1.81 – 1.13 (m, 24H), 0.99 (t, 3H, *J* = 6.4 Hz). ^{13}C NMR (100 MHz, $CDCl_3$, ppm) δ: 80.2, 80.0, 63.0, 32.7, 31.2, 29.5, 29.4, 29.1, 28.8, 25.7, 21.9, 18.7, 18.4, 13.6. MS (EI, 70eV): *m/z* (%):41 (34), 43 (14), 53 (9), 54 (43), 55 (52), 67 (66), 68 (28), 69 (20), 79 (22), 80 (10), 81 (100), 82 (33), 83 (10), 93 (13), 95 (43), 96 (95), 97 (12), 109 (11), 110 (24), 121 (2), 135 (2), 266 (1).

*(Z)-octadec-13-en-1-ol (**25**).* A mixture of **24** (50 mg, 0.188 mmol), methanol (2.0 mL), Pd/$CaCO_3$ (5 mg), and quinoline (5 mg) was subjected to hydrogen atmosphere (10 atm) at room temperature in a Parr® reactor (model 3910). After 2 h, the mixture was filtered and the solvent removed under reduced pressure. The residue was dissolved in hexane, and the organic layer washed with distilled water and dried over anhydrous Na_2SO_4. The solvent was evaporated under vacuum, and the crude product purified by silica gel column chromatography (hexane/ethyl acetate 8:2) affording compound **25** in 85% yield.[3] MS (EI, 70eV): *m/z* (%): 41 (47), 43 (23), 54 (25), 55 (100), 56 (19), 57 (18), 67

(51), 68 (39), 69 (50), 70 (12), 71 (7), 81 (59), 82 (78), 83 (35), 95 (43), 96 (64), 97 (24), 109 (20), 110 (21), 123 (12), 124 (13), 138 (8), 250 (5).

*(Z)-octadec-13-enal (**1**).* This compound was produced in 90% yield following a method analogous to that used to prepare compound **4**, starting from **25** (10 mg, 37,9 µmol). ^{1}H NMR (400 MHz, $CDCl_3$, ppm) δ: 9.76 (t, 1H, *J* = 1.9 Hz), 5.35 (ddd, 2H, *J* = 5.6 Hz, *J* = 4.4 Hz, *J* = 1.1 Hz), 2.41 (td, 2H, *J* = 7.4 Hz, *J* = 1.9 Hz), 2.35 (t, 2H, *J* = 7.5 Hz), 1.80-1.26 (m, 24H), 0.90 (m, 3H). ^{13}C NMR (100 MHz, $CDCl_3$, ppm) δ: 203.0, 129.9, 129.9, 43.9, 32.0, 29.8, 29.7, 29.7, 29.6, 29.5, 29.4, 29.3, 29.2, 27.2, 26.9, 22.4, 22.1, 14.0. MS (EI, 70eV): *m/z* (%): 41 (53), 43 (27), 54 (17), 55 (100), 56 (24), 57 (25), 67 (42), 68 (20), 69 (53), 70 (24), 71 (11), 79 (10), 81 (39), 82 (34), 83 (36), 84 (18), 93 (9), 95 (33), 96 (30), 97 (29), 98 (33), 109 (15), 110 (11), 111 (16), 121 (15), 135 (8), 248 (4), 266 (1).

*(Z)-octadec-13-en-1-yl acetate (**6**).* This compound was produced in 96% yield following a method analogous to that used to prepare compound **9**, starting from **25** (10 mg, 37,9 µmol). ^{1}H NMR (400 MHz, $CDCl_3$, ppm) δ: 5.35 (dd, 2H, *J* = 5.5 Hz, *J* = 4.3 Hz), 4.05 (t, 2H, *J* = 6.8 Hz), 2.04 (s, 3H), 1.64 – 1.59 (m, 2H), 1.34 – 1.21 (m, 24H), 0.94 – 0.88 (m, 3H). ^{13}C NMR (100 MHz, $CDCl_3$, ppm) δ: 171.3, 129.9, 129.9, 64.7, 32.0, 29.8, 29.7, 29.7, 29.6, 29.6, 29.5, 29.3, 29.3, 28.6, 27.2, 26.9, 25.9, 22.4, 21.0, 14.1. MS (EI, 70eV): *m/z* (%): 41 (52), 42 (10), 43 (88), 54 (30), 55 (100), 56 (20), 57 (65), 61 (13), 67 (57), 68 (41), 69 (48), 70 (13), 71 (41), 81 (70), 82 (93), 83 (39), 85 (29), 95 (50), 96 (84), 97 (31), 99 (12), 109 (24), 110 (30), 111 (11), 123 (16), 137 (10), 138 (14), 250 (15), 310 (1).

References

1. Zarbin, P. H. G.; Lorini, L. M.; Ambrogi, B. G.; Vidal, D. M.; Lima, E. R., Sex pheromone of Lonomia obliqua: Daily rhythm of production, identification, and synthesis. *J. Chem. Ecol.* **2007**, *33*, 555-565.
2. Mori, K., New synthesis of (11 Z, 13 Z)-11, 13-hexadecadienal, the female sex pheromone of the navel orangeworm. *Bioscience, biotechnology, and biochemistry* **2009**, *73*, 2727-2730.
3. Overman, L.; Brown, M.; Mccann, S., 4-(TRIMETHYLSILYL)-3-BUTEN-1-OL. *Org Synth* **1993**, *8*, 609-610.

4. NMR SPECTRA OF SYNTHETIC COMPOUNDS

12-bromododecan-1-ol (***13***)

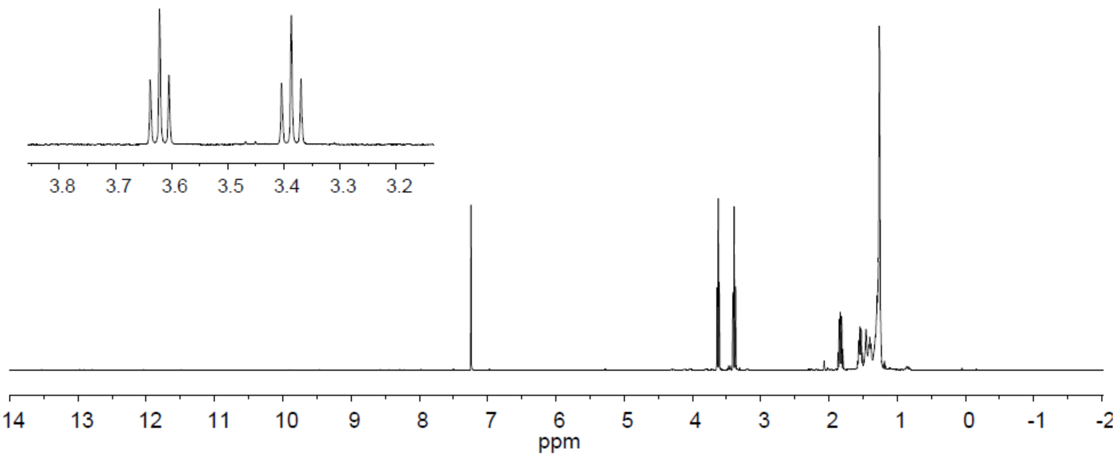

*^{1}H NMR Spectrum for compound **13.***

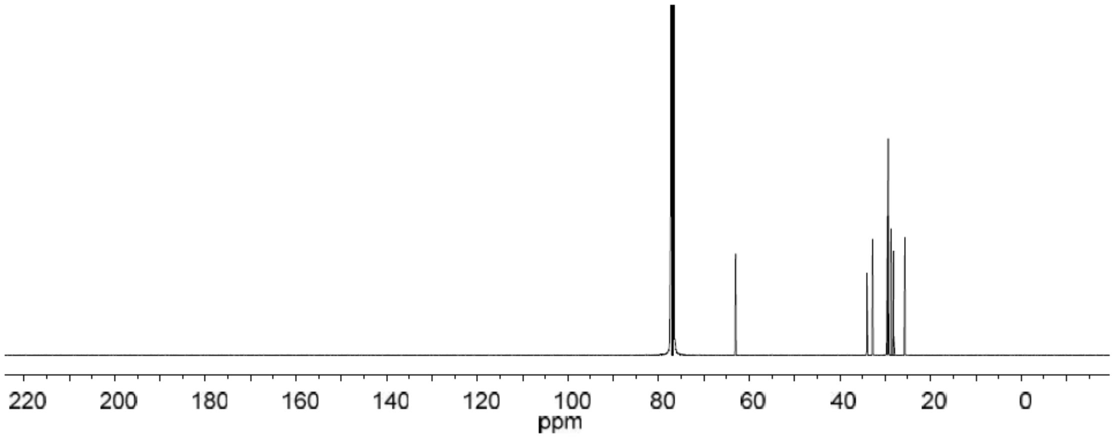

*^{13}C NMR Spectrum for compound **13.***

*2-((12-bromododecyl)oxy)tetrahydro-2H-pyran (**14**)*

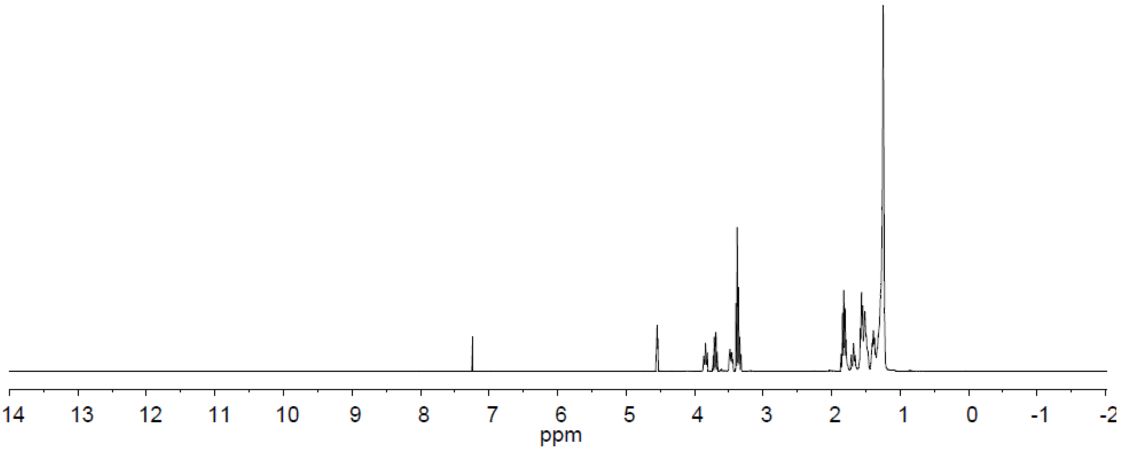

*^{1}H NMR Spectrum for compound **14.***

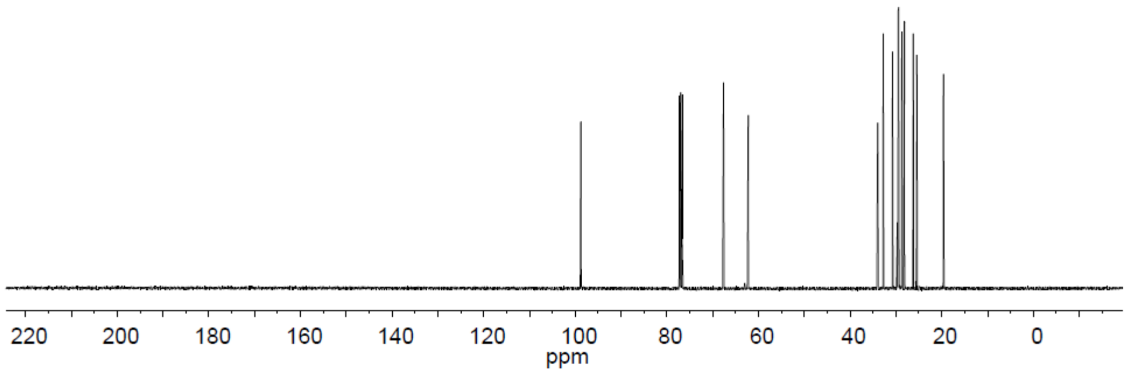

*^{13}C NMR Spectrum for compound **14.***

*2-(tetradec-13-yn-1-yloxy)tetrahydro-2H-pyran (**15**)*

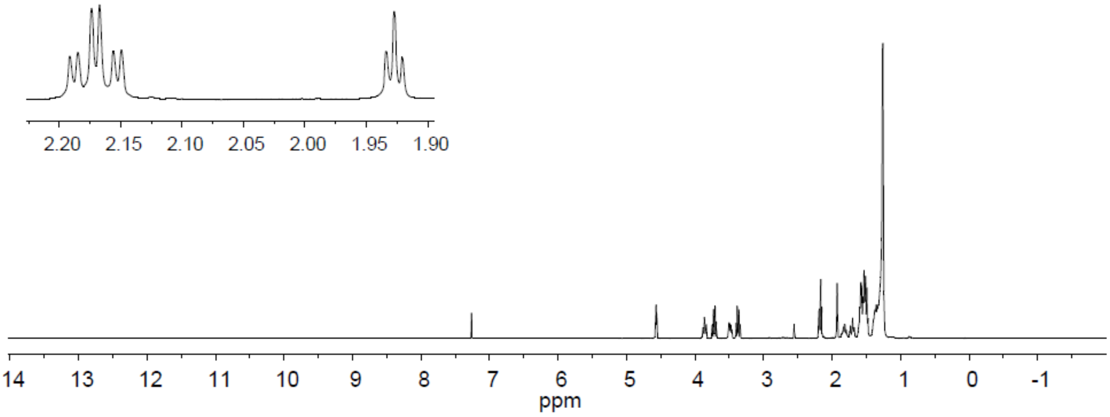

*^{1}H NMR Spectrum for compound **15.***

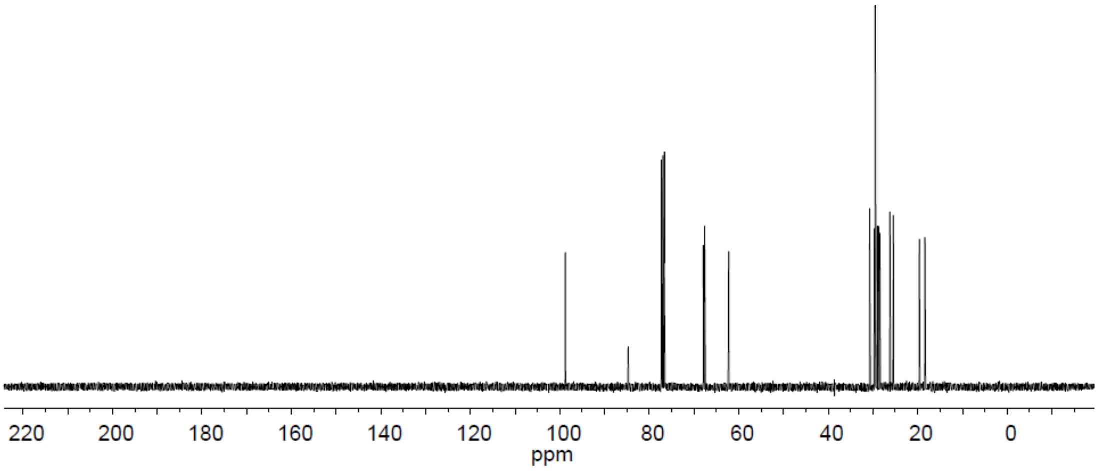

*^{13}C NMR Spectrum for compound **15.***

*(Z)-2-(octadec-15-en-13-yn-1-yloxy)tetrahydro-2H-pyran (**19**)*

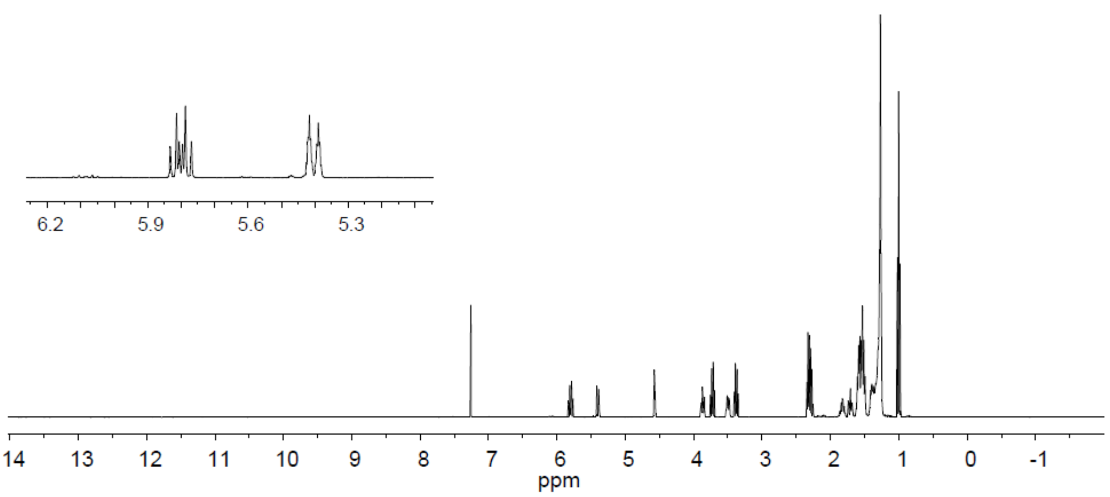

*^{1}H NMR Spectrum for compound **19.***

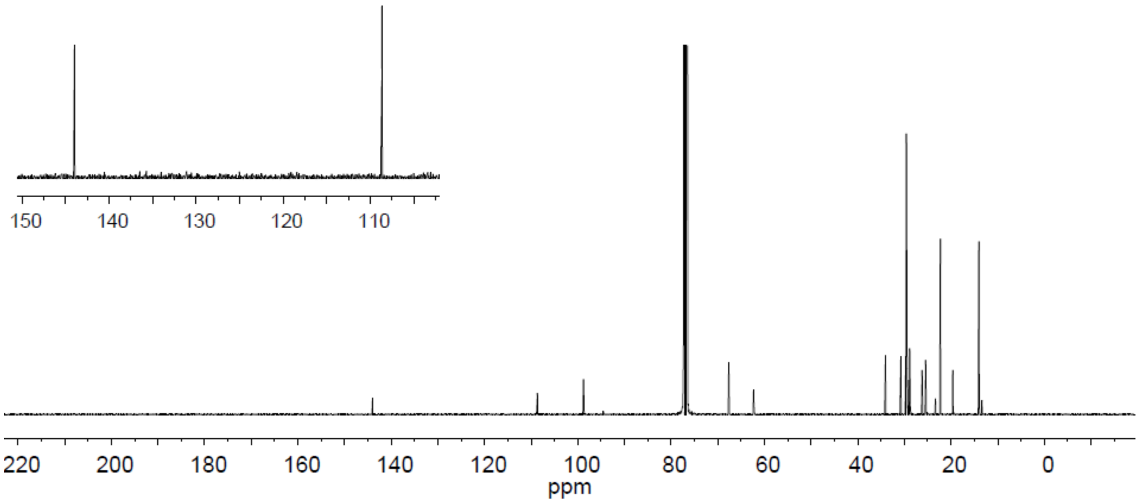

*^{13}C NMR Spectrum for compound **19.***

2-((13Z,15Z)-octadeca-13,15-dien-1-yloxy)tetrahydro-2H-pyran (**20**)

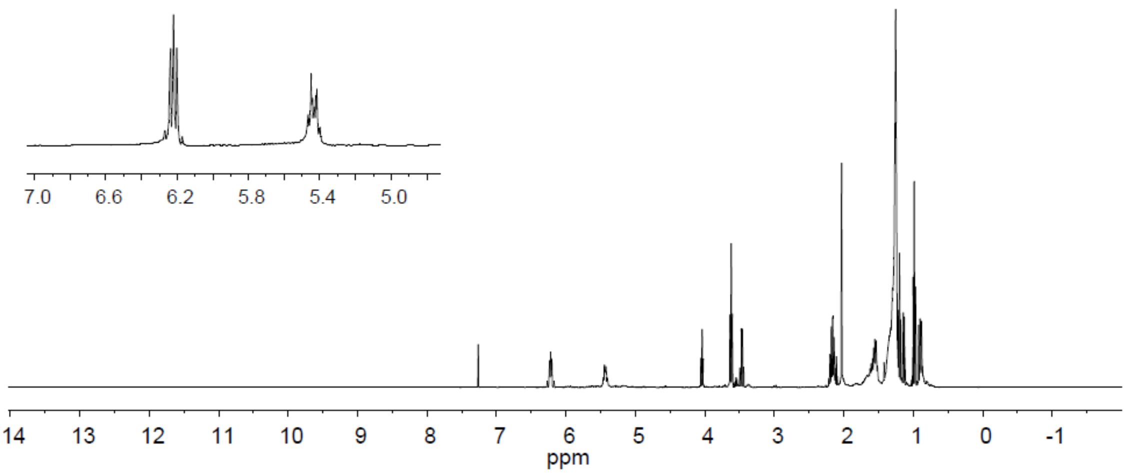

^{1}H NMR Spectrum for compound **20.**

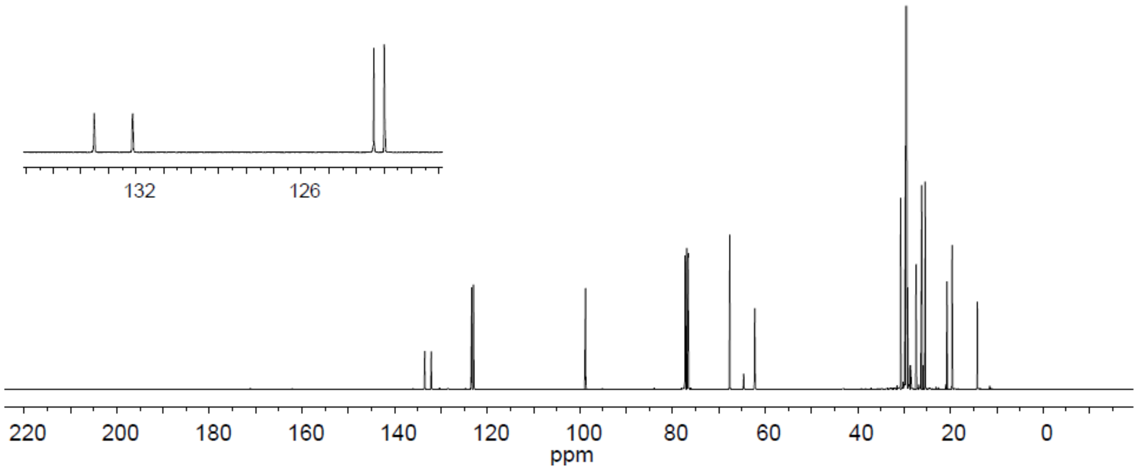

^{13}C NMR Spectrum for compound **20.**

*(13Z,15Z)-octadeca-13,15-dien-1-ol (**5**)*

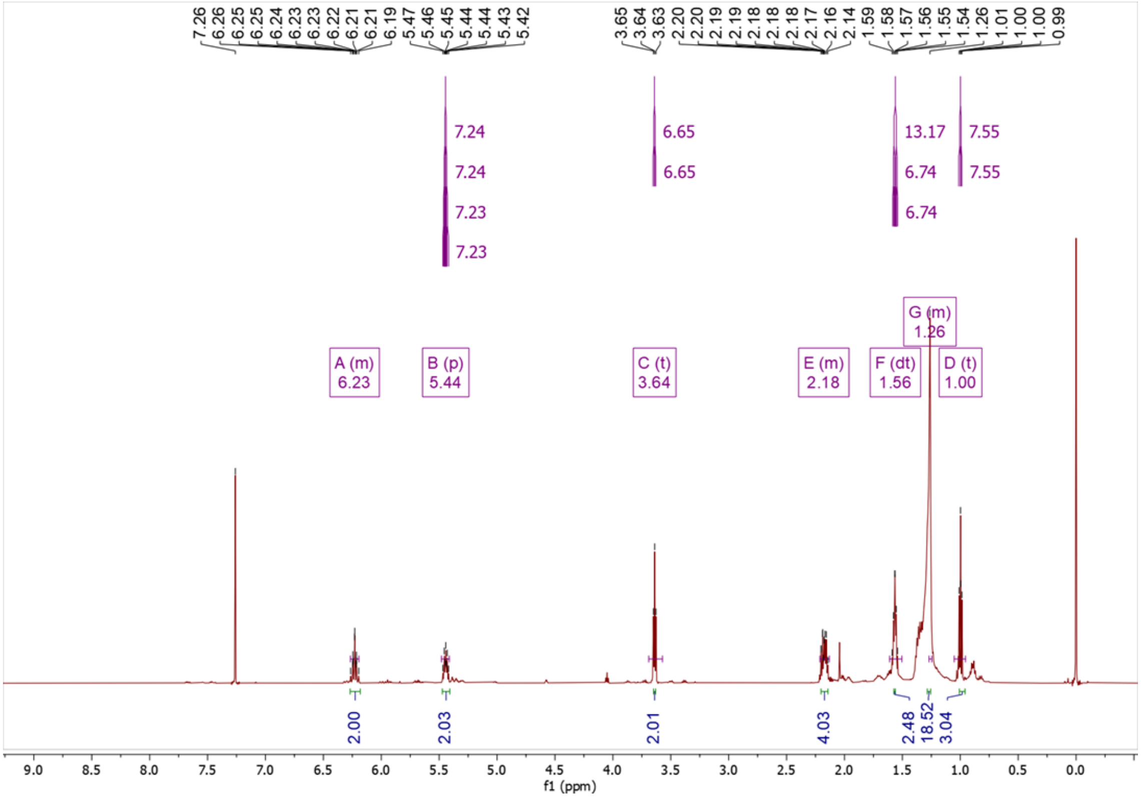

*^{1}H NMR Spectrum for compound **5.***

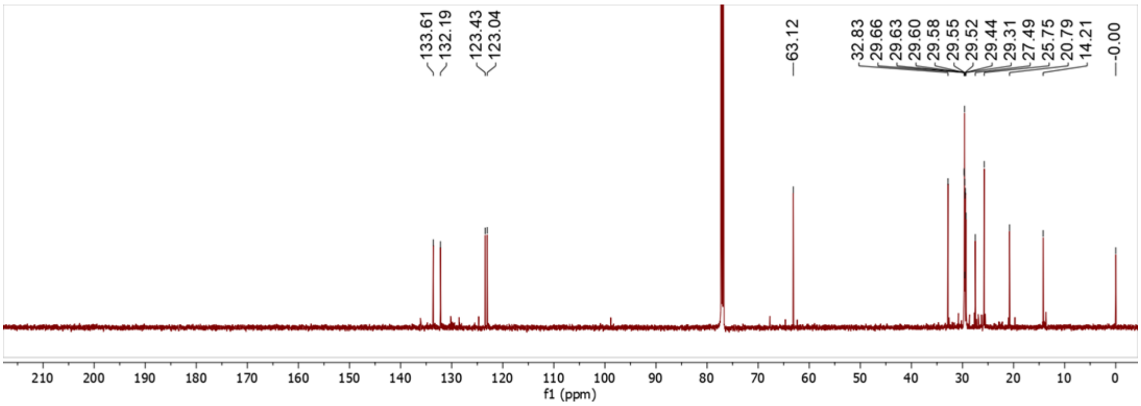

*^{13}C NMR Spectrum for compound **5.***

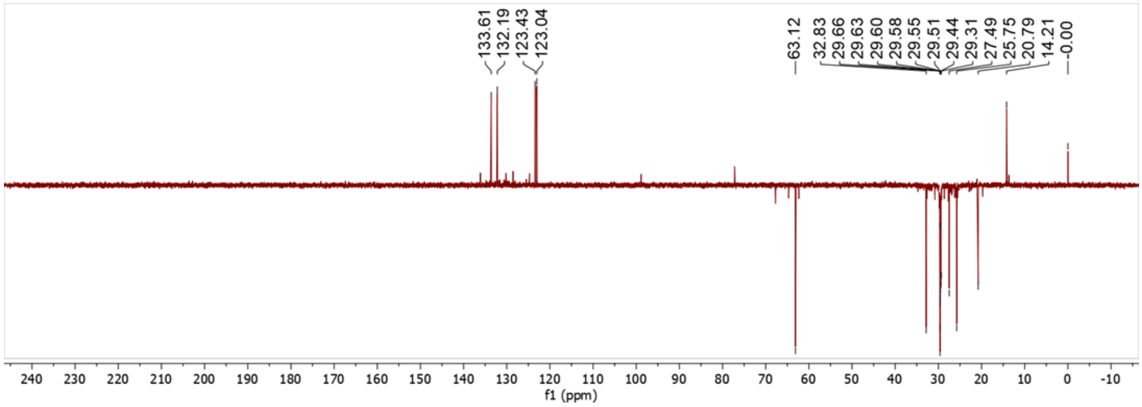

DEPT-135 NMR Spectrum for compound ***5****.*

*(13Z,15Z)-octadeca-13,15-dienal (**4**)*

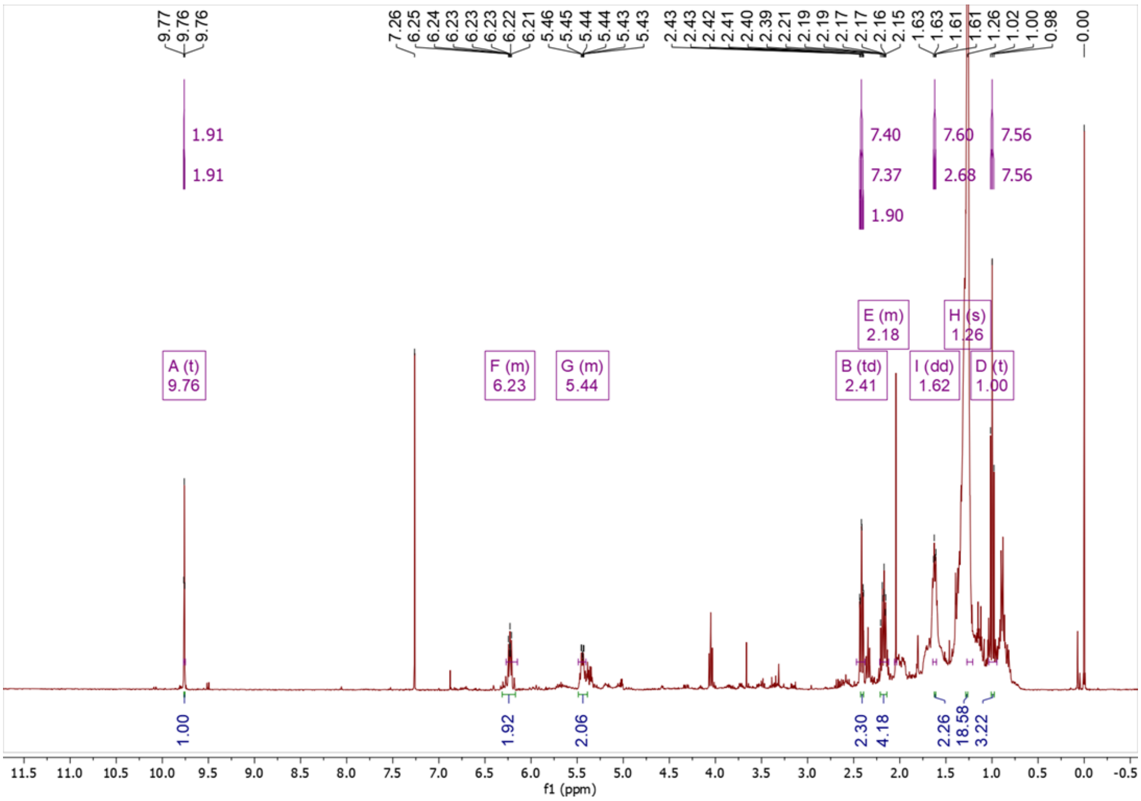

*^{1}H NMR Spectrum for compound **4**.*

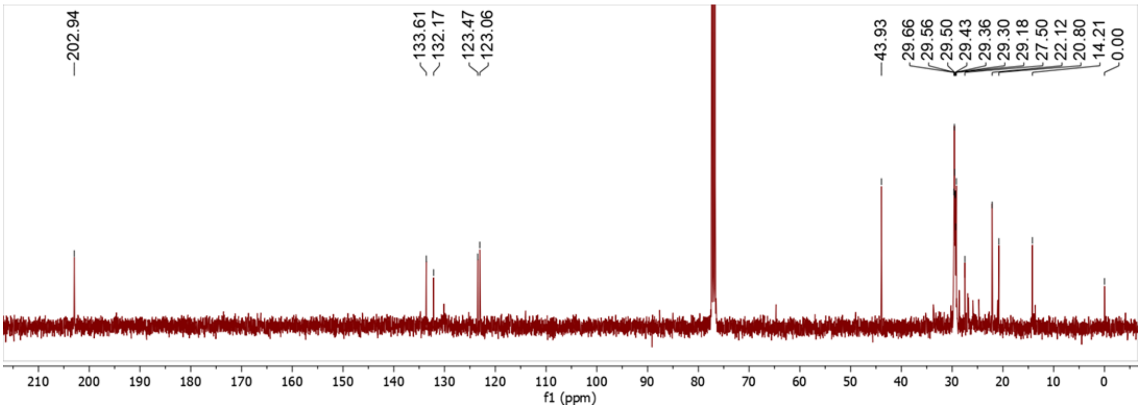

*^{13}C NMR Spectrum for compound **4**.*

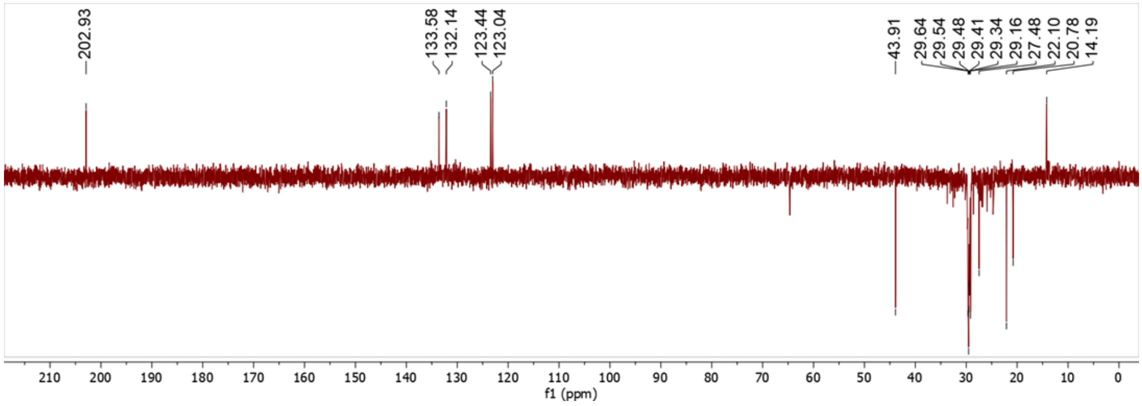

*DEPT-135 NMR Spectrum for compound **5**.*

*(13Z,15Z)-octadeca-13,15-dien-1-yl acetate (**9**)*

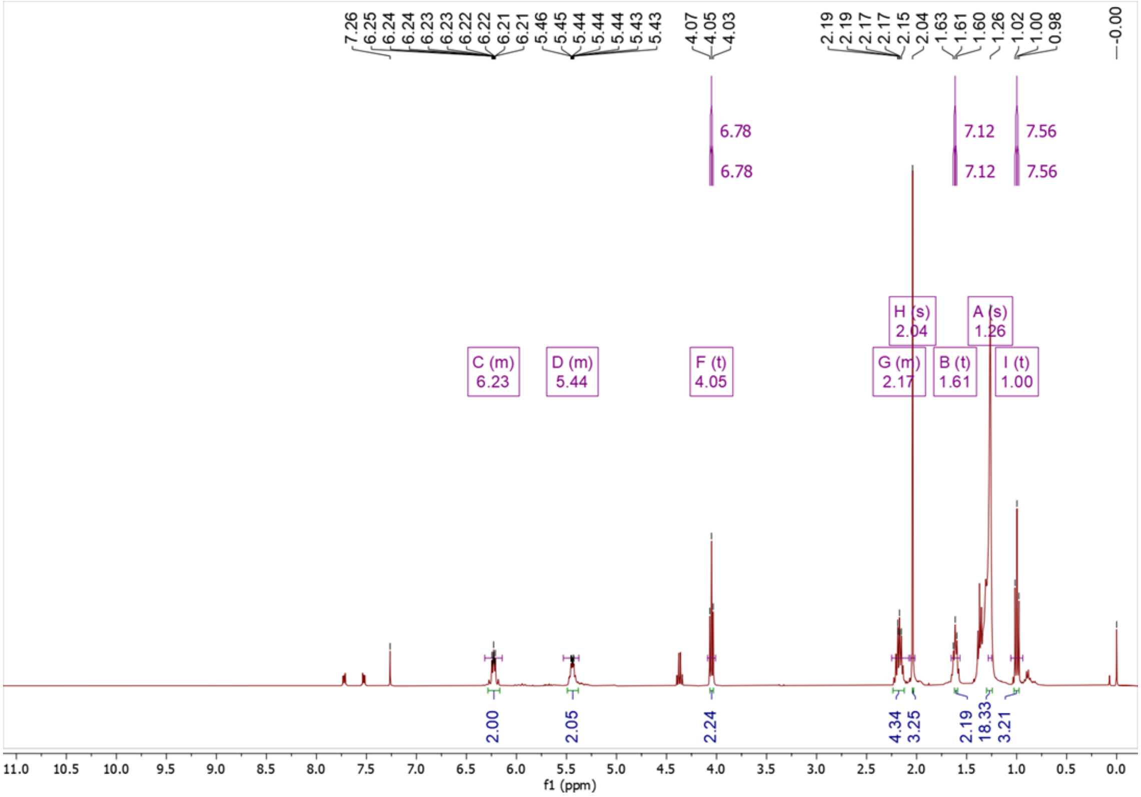

*^{1}H NMR Spectrum for compound **9**.*

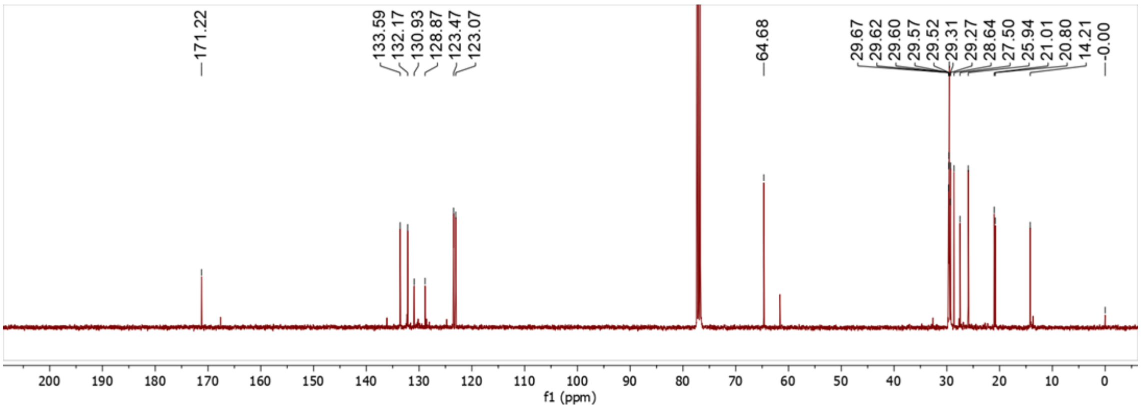

*^{13}C NMR Spectrum for compound **9**.*

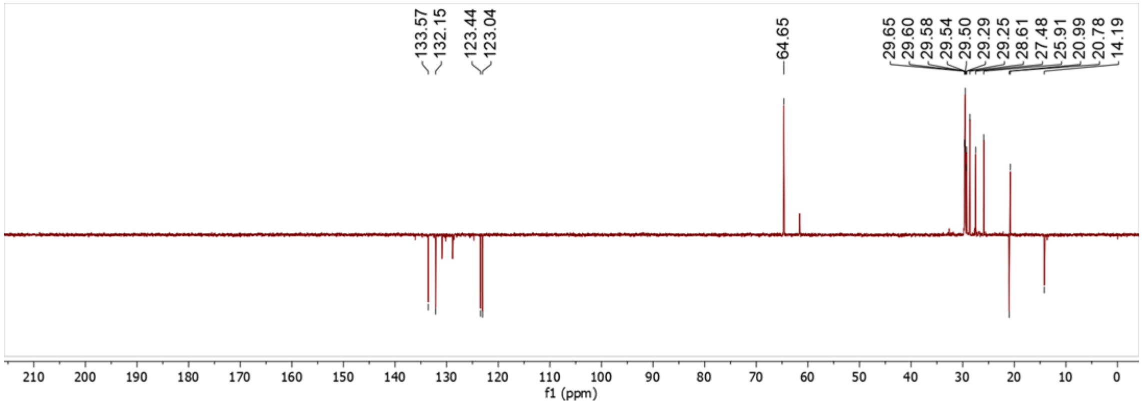

*DEPT-135 NMR Spectrum for compound **5**.*

*(Z)-1-iodobut-1-ene (**18**)*

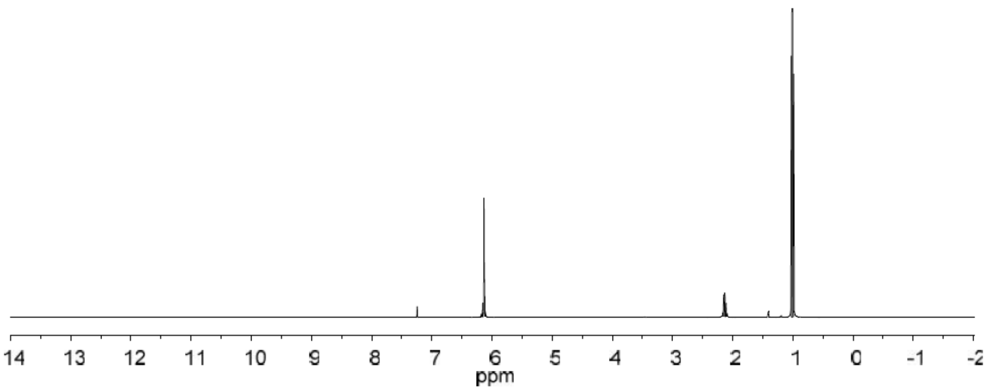

*^{1}H NMR Spectrum for compound **18.***

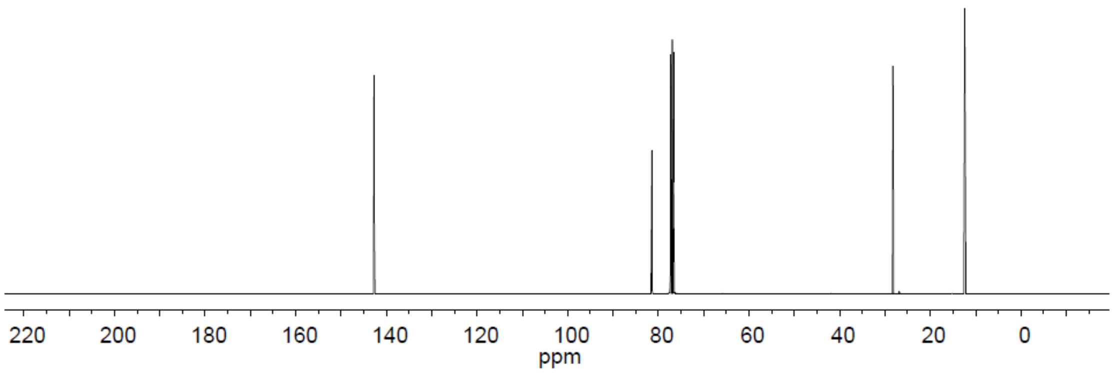

*^{13}C NMR Spectrum for compound **18.***

*octadec-13-yn-1-ol (**23**)*

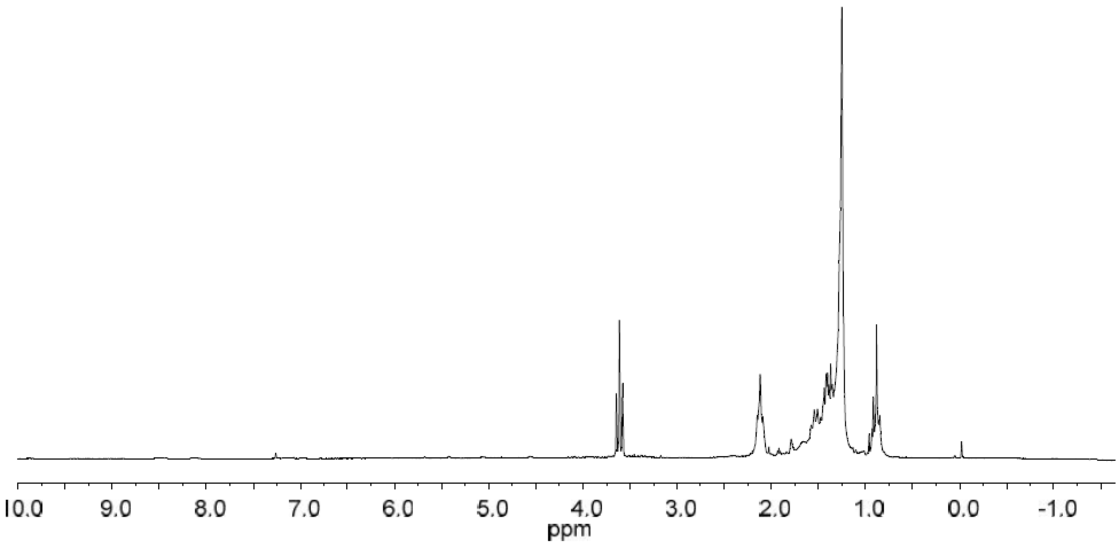

*^{1}H NMR Spectrum for compound **23.***

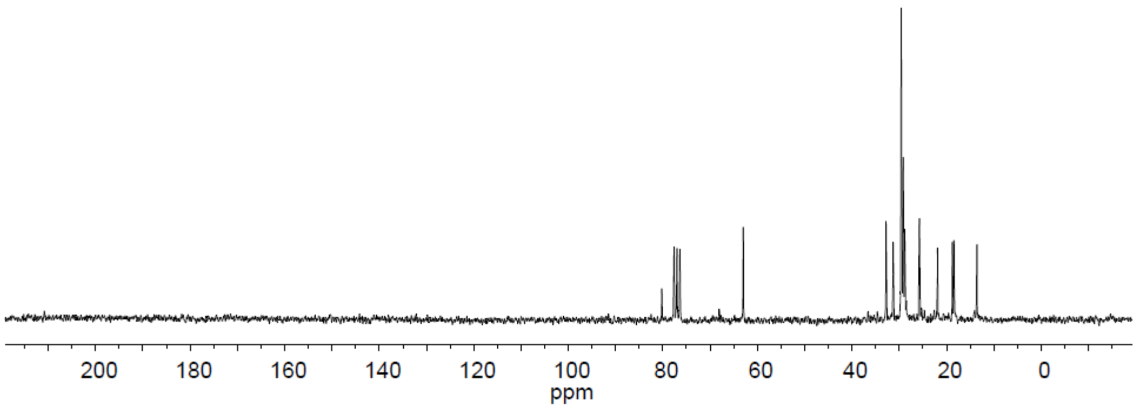

*^{13}C NMR Spectrum for compound **23.***

*(Z)-octadec-13-enal (**1**)*

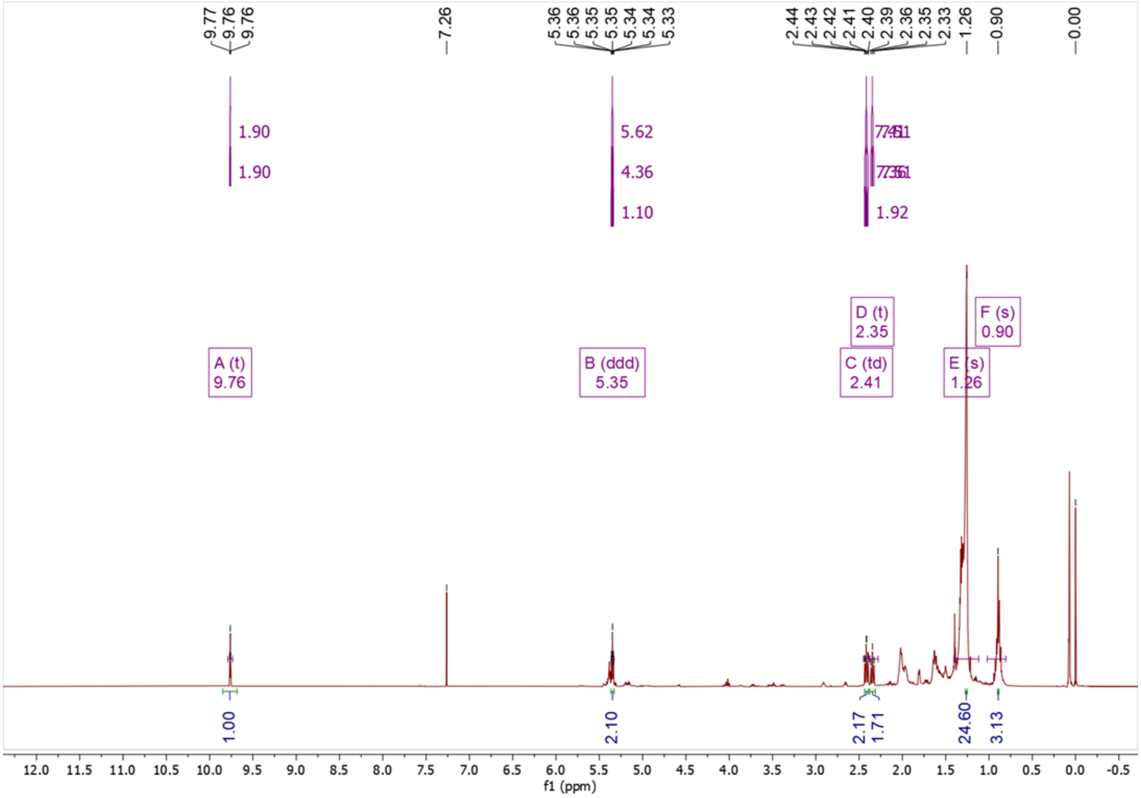

*^{1}H NMR Spectrum for compound **1.***

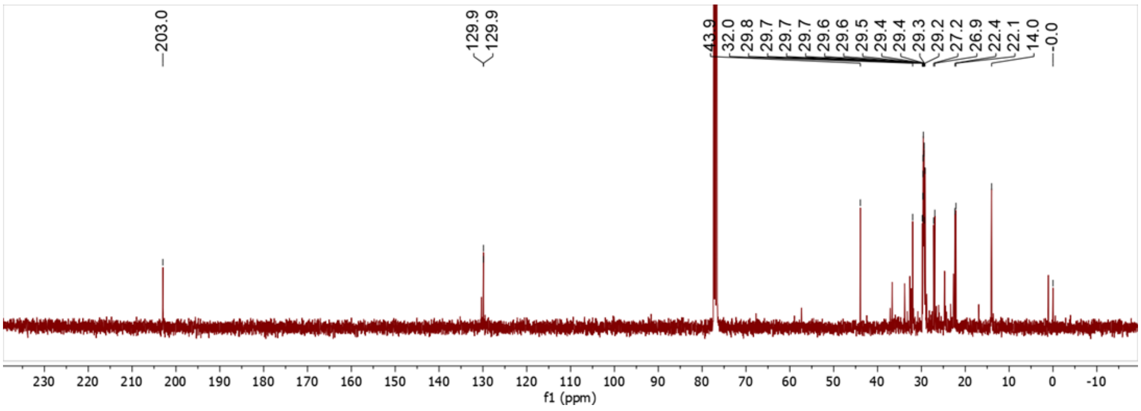

*^{13}C NMR Spectrum for compound **1.***

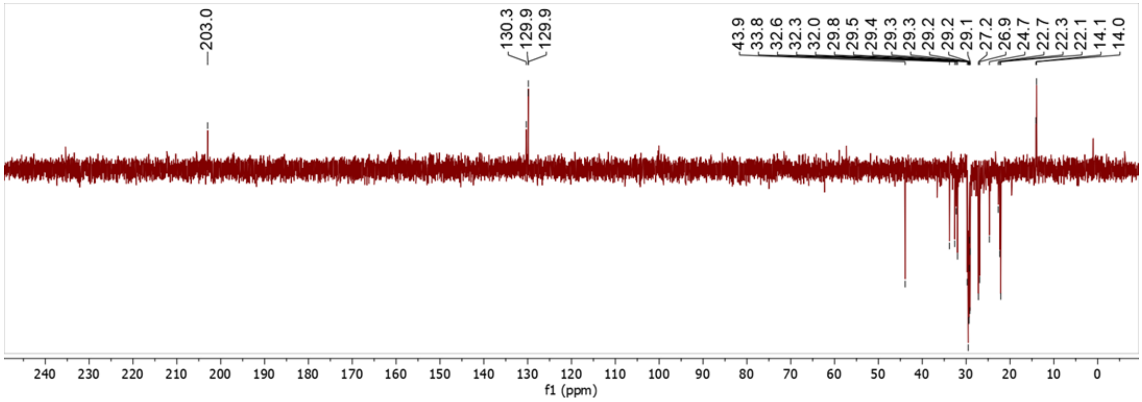

*DEPT-135 NMR Spectrum for compound **5**.*

*(Z)-octadec-13-en-1-yl acetate (**6**)*

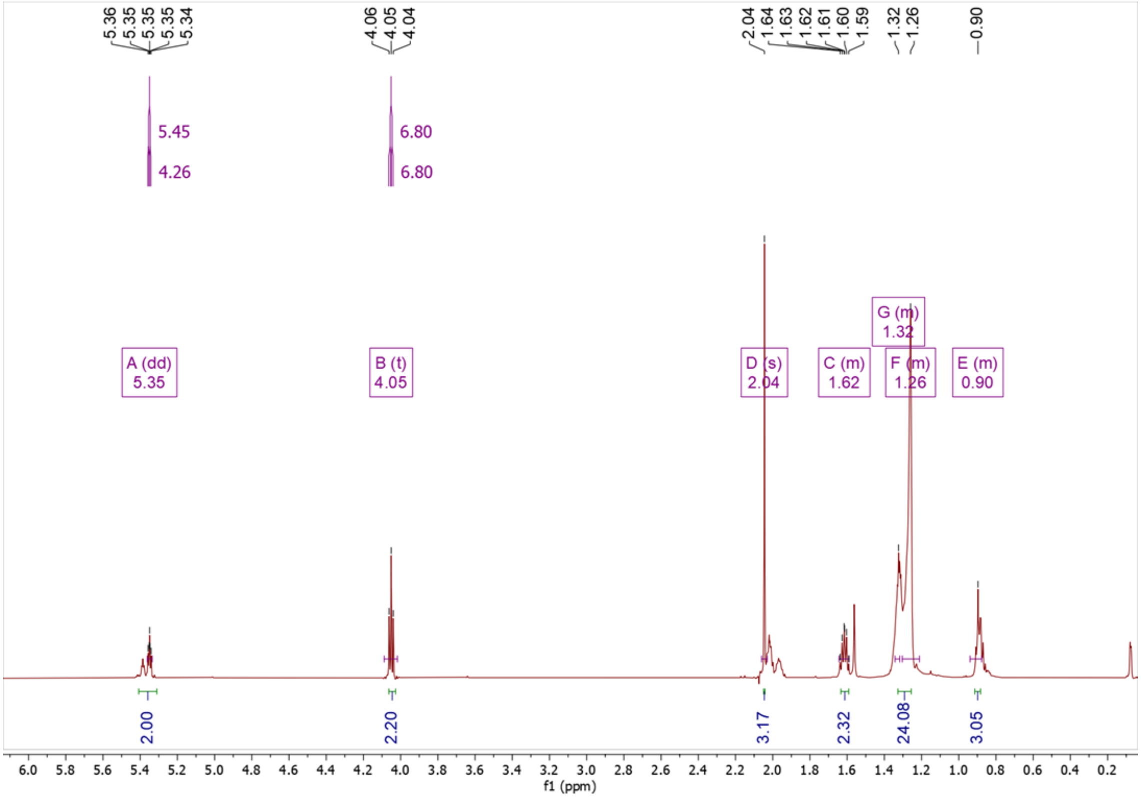

*^{1}H NMR Spectrum for compound **6**.*

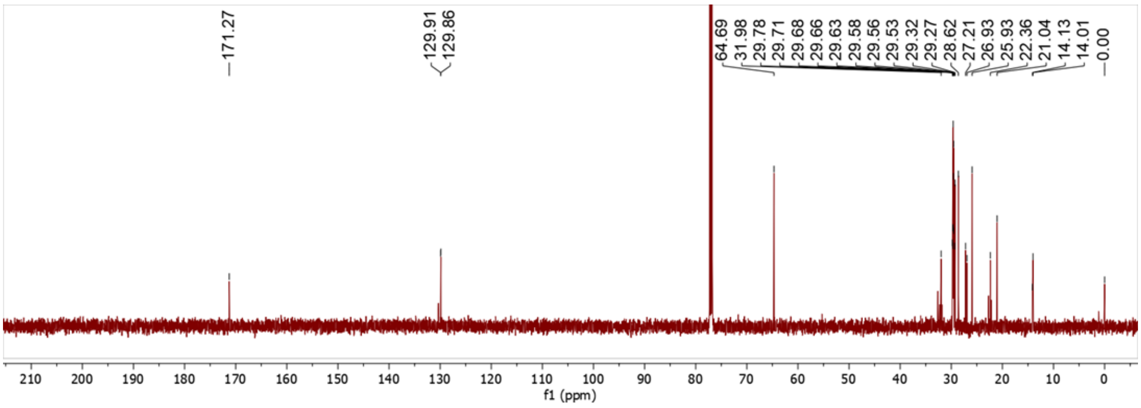

*^{13}C NMR Spectrum for compound **6.***

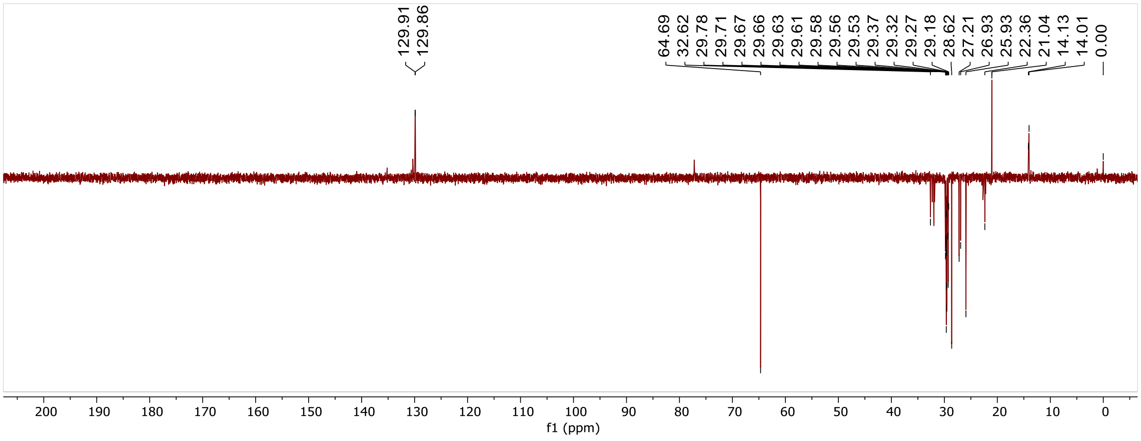

*DEPT-135 NMR Spectrum for compound **5**.*
